# Profiling Intact Glycosphingolipids with Automated Structural Annotation and Quantitation from Human Samples with Nanoflow Liquid Chromatography Mass Spectrometry

**DOI:** 10.1021/acs.analchem.4c00077

**Published:** 2024-04-02

**Authors:** Ryan L. Schindler, Armin Oloumi, Jennyfer Tena, Michael Russelle S. Alvarez, Yiyun Liu, Sheryl Grijaldo, Mariana Barboza, Lee-Way Jin, Angela M. Zivkovic, Carlito B. Lebrilla

**Affiliations:** † Department of Chemistry, University of California, Davis, Davis, California 95616, United States; ‡ Innovation Institute for Food and Health, University of California, Davis, Davis, California 95616, United States; § Department of Pathology and Laboratory Medicine, University of California Davis Medical Center, Sacramento, California 95817, United States; ∥ Department of Nutrition, University of California, Davis, Davis, California 95616, United States

## Abstract

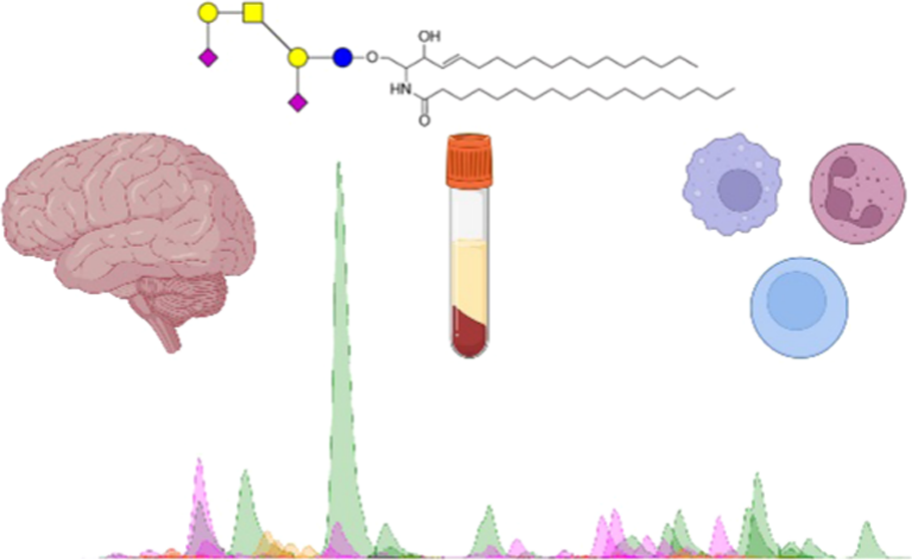

Sphingolipids are an essential subset of bioactive lipids found in most eukaryotic cells that contribute to membrane biophysical properties and are involved in cellular differentiation, recognition, and mediating interactions. The described nanoHPLC-ESI-Q/ToF methodology utilizes known biosynthetic pathways, accurate mass detection, optimized collision-induced disassociation, and a robust nanoflow chromatographic separation for the analysis of intact sphingolipids found in human tissue, cells, and serum. The methodology was developed and validated with an emphasis on addressing the common issues experienced in profiling these amphipathic lipids, which are part of the glycocalyx and lipidome. The high sensitivity obtained using nanorange flow rates with robust chromatographic reproducibility over a wide range of concentrations and injection volumes results in confident identifications for profiling these low-abundant biomolecules.

## Introduction

**Figure 1:**
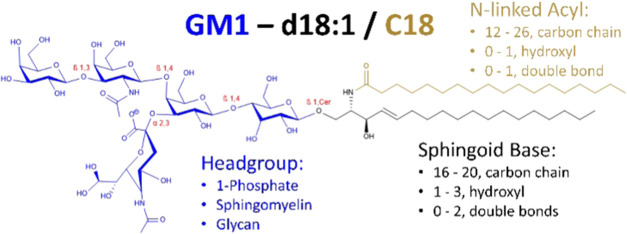
Molecular structure of GM1_a_ and summary of the structural diversity of human sphingolipids. The head groups are drawn in blue, and the sphingoid base is in black.

Sphingolipids are a class of amphipathic lipids found primarily in the outer membranes of eukaryotic cells.^1^ Since their discovery in 1884,^2^ researchers have explored the intricate degree of structural and functional diversity associated with this class of biomolecules. Their characterizing structural feature is the comprisal of a sphingoid base commonly referred to as a long-chain base (LCB). The addition of an N-linked acyl group forms a two-tailed lipid backbone referred to as a ceramide. Further derivatization is observed with the incorporation of a variety of different polar headgroups such as phosphatidylcholine, monosaccharides, as well as complex oligosaccharides. An example glycosphingolipid, GM1_a_, is depicted in Figure 1, including other possible headgroups and lipid compositions. These molecules contribute to membrane biophysical properties,^3^ mediate cellular interactions,^4−8^ and are involved in signaling,^9^ each attributed to the structural features of both the lipid ceramide and polar moiety.^10^ Sphingolipids have also been identified for their role in pathology where aberrant structures or abundances are observed.^11−13^

The consistent structural features found in the human sphingolipidome are attributed to the specificity of the enzymes involved in the biosynthetic pathways. De novo synthesis occurs in the endoplasmic reticulum producing the two-tailed ceramides with sphingosine (d18:1) being the most common sphingoid base but minor species such as Dihydroceramide (d18:0), 4-Hydroxydihydrosphinganine (t18:0), 6-Hydroxysphingosine (t18:1), and 4t,14c-Sphingediene (d18:2) are also present.^14−19^ Although the number of theoretically possible lipid structures is calculated to be over 4000 different species, roughly 500 unique ceramide structures have been discovered in humans.^9^ After ceramide synthesis, these lipids are translocated to the Golgi where headgroups are incorporated to the C1 position to form species such as ceramide-1-phosphate (1P-), sphingomyelin (SM-), Cerebrosides (Hex-), Sulfatides (SHex-, SLac-), and complex glycosphingolipids. The enzymatic pathways have been well established for 1P-, SM-, cerebrosides, and sulfatides, as a limited number of enzymes are involved. However, in the case of complex glycosphingolipids, the pathway to the finalized structure is more obscured. The complexity stems from their untemplated construction involving numerous glycosyltransferases with overlapping specificities. The activity of these enzymes is dependent on both localization in the ER and substrate availability. Complex oligosaccharides have been categorized by seven possible core structures; gala-, ganglio-, globo-, isoglobo-, lacto-, neolacto-, and muco-series^1^ where the tendency to express a specific core is dependent on the cell type. To date, roughly 450 unique glycan head groups have been discovered, a number which increases when considering likely intermediates and possible modifications such as lactone rings and acetylation.^1^ When considering the structural diversity of these molecules intact, the theoretical compound list includes over 200,000 unique species.

The structural diversity and relatively low abundance of these compounds within the overall lipid profile of a cell have made comprehensive analysis challenging. Previous works have used several techniques to elucidate the structural features of both the lipid^20−24^ and oligosaccharide headgroups^25^ through a combination of analytical and biochemical methods. Significant collaborative efforts have been made to assist in further research of these molecules. LIPID MAPS (https://www.lipidmaps.org/) includes a database of all previously discovered ceramide species as well as SphinGOMAP (https://sphingolab.biology.gatech.edu/), which has documented the complementary complex oligosaccharide headgroups.

Historically, analytical methodologies to profile sphingolipids used fluorophore-labeled monoclonal antibodies that bind to specific glycan structures^13^ or endoglycosylceramidase, which hydrolyzes the bond between the oligosaccharide and the ceramide.^26^ Both workflows provided the initial understanding of sphingolipid molecular structures but lacked information for the intact molecules. Modern techniques for sphingolipid profiling and quantitation employ high-performance liquid chromatography (HPLC) for the separation of these intact compounds coupled with accurate mass spectrometry (MS) detection.^27^

In this study, we developed a robust and reproducible method for quantitatively profiling intact glycosphingolipids (GSLs) with automated compound identification. In this work, the nomenclature follows the same convention commonly used based on IUPAC-IBU recommendations.^28^ The method employed nanoflow reverse-phase high-performance liquid chromatography and quadrupole time-of-flight mass spectrometry (nRP-HPLC-Q/ToF) to separate and detect GSLs from biological samples effectively. Compound identification is key and facilitated by using a combination of biological knowledge, accurate mass detection, collision-induced disassociation, and retention times to assign molecular structures instantaneously. This process is semiautomated with the utilization of Agilent’s Personal Compound Database and Library software (PCDL), which drastically reduces false-positive identifications by as much as 50%. Profiling can be accomplished in a fraction of the time with a high degree of confidence and minimal background knowledge of sphingolipids. The most common issues observed in sphingolipid analysis that led to unreliable data are carryover, in-source fragmentation, and false-positive identifications from isobaric lipid species. Improved chromatography and removal of carryover was accomplished by developing an online sample enrichment using a series of timed valve switches with a C-8 trap followed by separation on a C-18 column. Source conditions were optimized to maximize ion generation with minimal to zero in-source fragmentation with specification of the more susceptible compounds.

## Methods

### Materials and Chemicals

Sphingomyelin-d18:1/C18 (SM-d18:1/C18, Cat# 860586), Sphingomyelin-d18:1/C24:1 (SM-d18:1/C24:1, Cat# 860593), Glucose-d18:1/C24:1 (Glc-d18:1/C24:1, Cat# 860549), Sulfo-galactose-d18:1/C24:1 (SHex-d18:1/C24:1, Cat# 860571), GM1_a_-d18:1/C20 (Cat# 860588), GM3-d18:1/C18 (Cat# 860074), GD1_a_-d18:1/C18 (Cat# 860091), and GT1_b_-d18:1/C18 (Cat# 860089) standards were purchased from Avanti Polar Lipids (Alabaster, AL). Lymphoblast CESS cells (Cat# TIB-190) were obtained from the American Type Cell Culture (Manassas, VA). α2–3,6,8 Neuraminidase (Cat# P0720) was purchased from New England Biolabs (Ipswich, MA). Human serum (Cat# S7023), sucrose (Cat# S7903), potassium hydroxide (KOH, Cat# P5958), potassium chloride (KCl, Cat# P3911), ammonium acetate (NH_4_CH_3_CO_2_, Cat# 73594), sodium carbonate (Na_2_CO_3_, Cat# S5761), trichloromethane (CHCl_3_, Cat# CS10501), and protease inhibitor cocktail (Cat# 539137) were purchased from Sigma (St. Louis, MO). Fetal bovine serum (Cat# 16000–069), penicillin-streptomycin (Cat# 15140–122), 1 M HEPES (Cat# 15630080), methanol (MeOH, Optima LC/MS, Cat# A456–4), and isopropanol (IPA, Optima LC/MS, Cat# A461–4) were purchased from Thermo Fisher Scientific (Waltham, MA). C-8 SPE plate (100 mg, Cat# FNSC08.800) was purchased from Glygen. Glacial acetic acid (GAA, Cat# AC110) was purchased from Spectrum (New Brunswick, NJ). Formic acid (Optima LC/MS, Cat# A117–50) was purchased from Fisher Chemical (Hampton, NH).

### Brain Tissue

Human brain tissue was obtained through the University of California, Davis–Alzheimer’s Disease Center. The specific sample was taken from the lateral cerebellum of a single subject, age 93, with pathologically confirmed Alzheimer’s disease.

### Cell Culture

CESS lymphoblast (TIB-190) cells were cultured in RPMI-1640 Medium (ATCC, Cat# 30–2001) containing 10% (v/v) fetal bovine serum and 1% (V/V) penicillin-streptomycin in 75 mm^2^ culture dishes. The cells were maintained in a humidified incubator at 37 °C with 5% CO_2_ subcultured at 80% confluency for five passages and harvested at 80% confluency in the sixth passage.

### Standard Preparation

External standards SM-d18:1/C18, SM-d18:1/C24:1, Glc-d18:1/C24:1, SHex-d18:1/C24:1, and GM1_a_-d18:1/C18 were received as ammonium salts and diluted to 50 μM stock solutions in MeOH/IPA/water (2:8:1, v/v/v%). Further dilution used MeOH/water (1:1, v/v%). GM3-d18:1/C18 (100 μg/mL), GD1_a_-d18:1/C18 (100 μg/mL), and GT1_b_-d18:1/C18 (124 μg/mL) were received as MeOH solutions, and diluted in MeOH/water (1:1, v/v%).

### Sample Preparation: Tissue, Serum, and Cells

∼10^6^ cells, 1–100 mg of neural tissue, and 100 μL of serum were used to generate sample profiles. The tissue was weighed into 15 mL falcon tubes and diluted with a buffer consisting of 0.25 M sucrose, 20 mM HEPES adjusted to pH 7.4 with KOH, and a 1:100 protease inhibitor cocktail (1.2 mL for cells and 1.5 mL for tissue). Tissue samples were homogenized manually before lysis with μ-needle sonication (60 J for cells and 80 J for tissue samples).

The nuclear fraction was precipitated by centrifugation at 2000 RCF for 10 min. The supernatant was transferred and ultracentrifuged at 200k RCF for 30 min at 4 °C to form a membrane pellet. After removing the supernatant, samples were diluted with 0.2 M Na_2_CO_3_ (0.5 mL for cells and 1.0 mL for tissue) and ultracentrifuged to remove membrane-associated proteins. The supernatant was removed, and samples were ultracentrifuged again with the same volume of water. After discarding the water, membrane lipids were dissolved using a modified Folch extraction of freshly prepared water/MeOH/CHCl_3_ (3:8:4, v/v/v%, 500 μL for cells and 800 μL for tissue/serum) and sonicated for 30 min. Samples were then centrifuged at 9000 RCF for 10 min to precipitate the membrane proteins, and the supernatant was collected. 100 μL of 0.1 M KCl was added to induce a liquid–liquid separation, the top layer (aqueous) was transferred and dried by vacuum centrifugation.

Sphingolipids were enriched with a 100 mg, C-8, 96-well SPE plate. Wells were first conditioned with 200 μL of MeOH/IPA (1:1, v/v%) and primed with 400 μL of water/MeOH (1:1, v/v%). Samples were reconstituted with 600 μL of water/MeOH and gravity-loaded. The flow-through was reloaded to ensure maximum recovery. 600 μL of water/MeOH was used to wash. Sphingolipids were eluted with 200 μL of the MeOH/IPA and then dried. Condition, prime, wash, and elution steps used centrifugation (100 RCF, 1 min).

Dried samples can be sealed and stored at −20 °C for several months until ready for analysis. Before analysis, samples were reconstituted in water/MeOH (1:1, v/v%) (20 μL for serum/cells and 0.25 mg/μL for tissue), transferred to autosampler vials, and stored in the 4 °C cooler for up to 7 days before injection.

### Neuraminidase Treatment

α2–3,6,8-Neuraminidase was used following the vendor’s recommendation and found to hydrolyze terminal sialic acid residues preferentially. Enzyme-treated samples required an additional sample injection using a unique instrumental method with an increased online enrichment step, increasing all gradient and valve switch time points by 9 min. Comparison of the reduction in initial signal and increase in resulting products from nontreated to treated samples allow determination of a- and b-series gangliosides. The crystal structure of *Clostridium perfringens* sialidase nanH was modeled using AlphaFold^29^ using the sequence information from UniProt.^30^ Glycolipid models were drawn using CHARMM-GUI.^31^ After modeling, the 3D structures of the sialidase enzyme and glycolipid substrates were minimized and prepared for in-silico docking experiments using Chimera.^32^ In silico docking, calculations were performed in PyRx^33^ using AutoDock VINA^34^ by defining a 24 × 25 × 56 Å^3^ search space enclosing the reported active site residues of the enzyme. After performing calculations, the models were visualized, and binding interactions were identified using Discovery Studio (Dassault Systems, 2020).

### Nanoflow HPLC-Q/TOF Methodology

Automated sample injection and data collection used an Agilent 1200 series nanoflow HPLC. Online sample enrichment used a Zorbax 300SB-C8 trap column, 0.3 ID × 5 mm, 5 μm particle size, 300 Å pore size (Agilent Technologies Inc., Cat# 5065–9914). The analytical separation was carried out on a Zorbax 300SB-C18 column, 0.075 ID × 150 mm, 3.5 μm particle size, 300 Å pore size (Agilent Technologies Inc., Cat# 5065–9911). The loading/washing pump was operated at 2.5 μL/min. Sample loading used 0.1% GAA and 20 mM NH_4_CH_3_CO_2_ in water/MeOH/IPA (40:50:10, v/v/v%). Sample washing (MP-W) used MeOH/IPA (1:1, v/v%). The gradient used was as follows: 0% MP-W from 0 to 20 min, increased to 99% at 25 min, held until 35 min, decreased back to 0% at 40 min, and held until 70 min. The analytical gradient pump used a flow rate of 0.3 μL/min. Mobile phase A (MP-A) used 0.1% GAA in 20 mM NH_4_CH_3_CO_2_ in MeOH/water (25:75, v/v%), and mobile phase B (MP-B) with a composition of 0.1% GAA in 20 mM NH_4_CH_3_CO_2_ in MeOH/IPA (75:25, v/v%). The timed composition changes are as follows: 76% MP-B from 0 to 20 min, a linear increase to 96% at 60 min, held until 62 min, decreased to 76% by 64 min, and held until 70 min. The C-8 trap column (left) was operated at 70 °C, and the C-18 analytical column (right) was operated at 60 °C. The trap and analytical columns have a working range of up to 90 °C for 2–5 pH. A 10 pt/2 ps μ-switching valve was configured for efficient enrichment, elution, and washing at low flow rates. Samples are enriched from 0 to 6 min (μ-valve 1 → 10). From 6 to 20 min, analytes are backflushed from the C-8 trap to C-18 analytical column under stepped isocratic conditions (μ-valve 1 → 2). From 20 to 70 min, the gradient, wash, and equilibration are carried out (μ-valve 1 → 10).

The analytical column was coupled to an orthogonal nanoESI source (Agilent Technologies, G1992A) and operated in positive ion mode with a 15 μm ID SilicaTip (New Objective). Precursor ion mass filtering, fragmentation, and detection were performed on a quadrupole time-of-flight mass spectrometer (Agilent Technologies, G6520A). Source conditions were optimized by direct infusion and used N_2_ drying gas at 325 °C with a flow rate of 3.0 L/min. The capillary voltage was 1300 V and adjusted during initial system conditioning for a corresponding current of 0.070 μA and stable spray throughout the gradient. The fragmentor, skimmer, and octopoleRF voltages were set to 150, 90, and 750 V, respectively. The quadrupole used automatic precursor ion selection with a mass range of 550–2000 *m*/*z* and an absolute threshold of 1000 counts, corresponding to roughly double the baseline noise. The preferred charge state was set to 2 > 1. Precursor ions were fragmented in an N_2_-filled chamber with collision-induced dissociation using an *m*/*z* dependent collision energy determined by linear interpolation with the equation . Active exclusion was enabled after collection of one MS_2_ spectrum and released after 1 min corresponding to approximately one-half the average peak width. The time-of-flight detector was operated to collect abundance and accurate mass for 100–2000 *m*/*z*. An internal reference mass of 1221.9 *m*/*z* (Agilent Technologies Inc., Cat# G1982–85001) was used for continuous mass correction (≤10 ppm). The HPLC modules, valve configuration, connecting capillaries, and source settings that were used are included in the Supporting Information.

### Data Analysis

Postacquisition compound identification and peak integration were completed using Agilent’s MassHunter Qualitative Analysis software version (B08.00) with the Find by Molecular Feature (FMF) algorithm using a CSV database of compounds including the molecular formula, retention time (optional), mass, name, and description. Verification assistance of the identified compounds used Agilent’s Personal Compound Database and Library (PCDL) software version (B08.00), where the identified compounds are compared and scored from a spectral library. Library search settings enabled screening and score adjustment with a precursor and fragment mass tolerance of 25 and 50 ppm, respectively. Each sample’s compound list was exported to individual CSV files and an in-house Python script was used to organize the data for analysis in Excel. The Python script is included as a Supporting file.

## Results and Discussion

### Nanoflow High-Pressure Liquid Chromatography–Mass Spectrometry Profile of Sphingolipids from Brain Tissue

**Figure 2:**
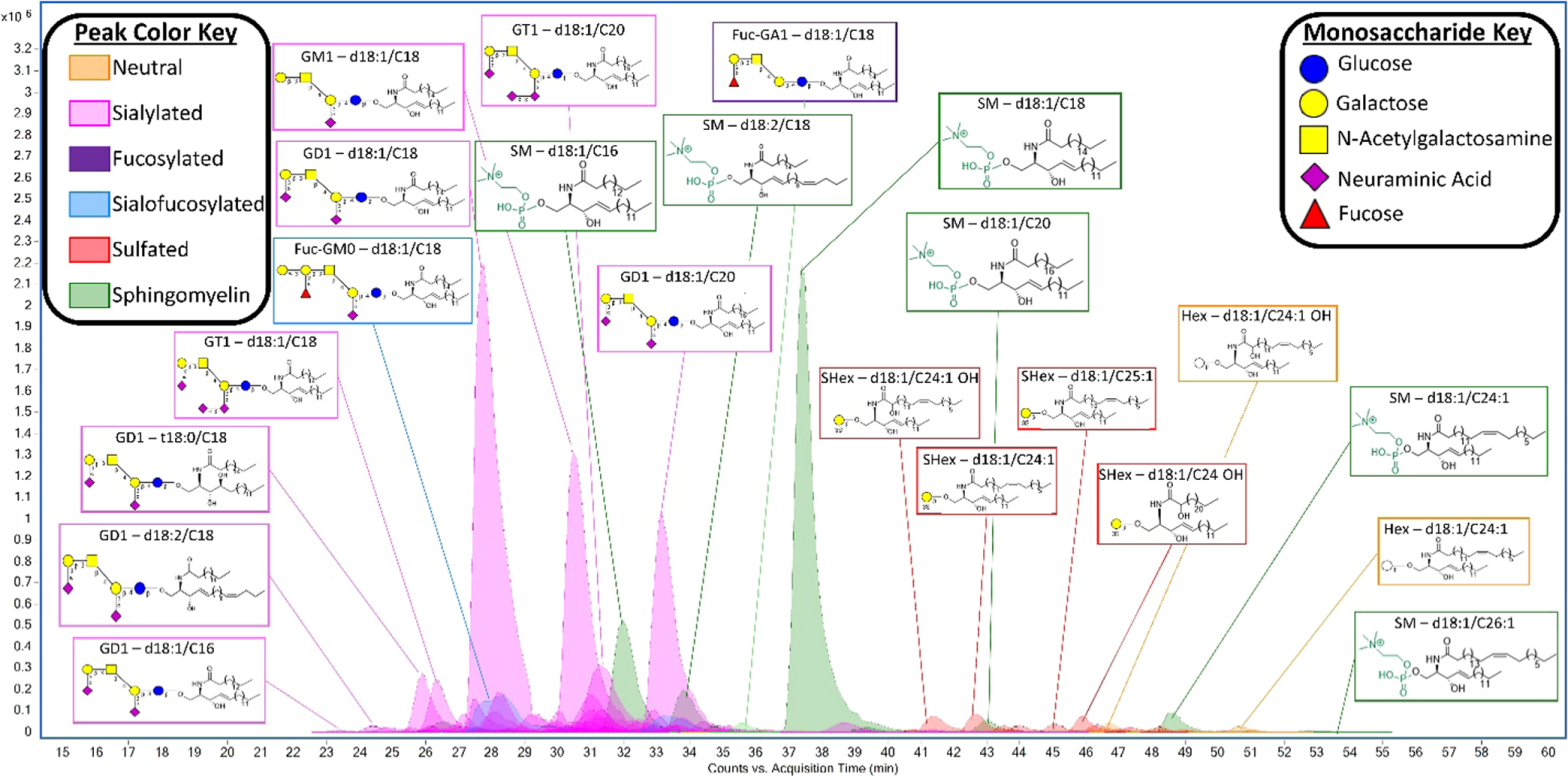
Example chromatogram of human brain tissue sphingolipid profile annotating 22 of 118 compounds found. Inset structures were assigned based on the methods described.

**Figure 3:**
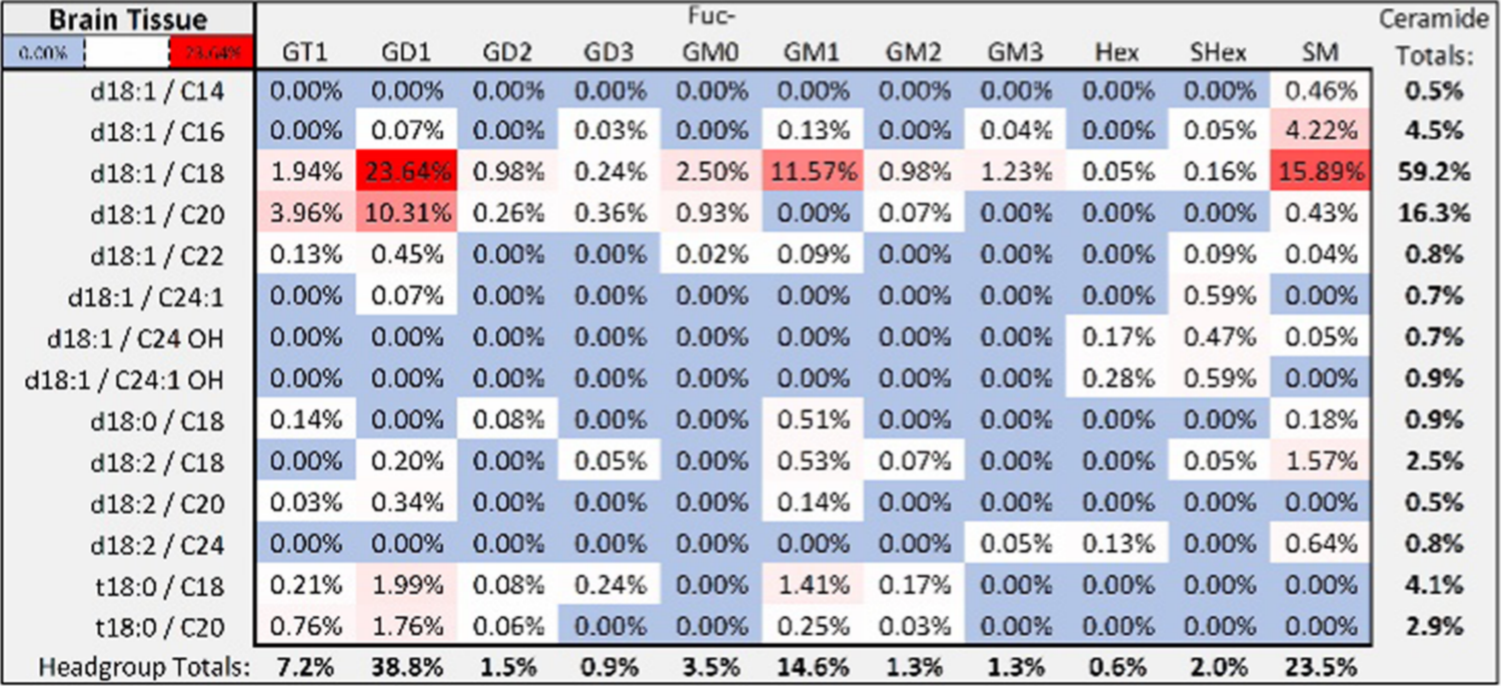
Heatmap summarizing the relative intensities of sphingolipids (≥0.01% relative abundance) in human brain tissue. The major products correspond to GM1, GD1, and SM.

A reverse-phase nanoflow HPLC-Q/ToF method was developed and employed to extensively profile the intact sphingolipids found in human neural tissue, serum, and a lymphoblast cell line. A representative chromatogram is depicted based on the reported method, where the major peaks are labeled with their representative structures (Figure 2). This tissue profile, from the lateral cerebellum, yielded 118 unique compounds varying in both headgroup and lipid structure. A summary of the structures present in the chromatogram with the respective relative abundances of ≥0.01% are summarized with a heatmap (Figure 3) using IUPAC-IUB nomenclature.^28^ The structural assignment is comprehensive and was determined using the methods described in greater detail below. The major sphingolipids observed were gangliosides GD1_a_, GD1_b_, and GM1_a_. These oligosaccharide headgroups are typically observed in the gray matter regions of the brain, which are primarily composed of neuronal cell bodies and their dendrites. We also observed gangliosides with up to four sialic acid residues (GQ1) as well as some fucosylation and galactose extension, which are rarely observed. The most abundant ganglioside-associated lipid was sphingosine (d18:1) with an N-linked acyl group of 18 and 20 carbons. The less abundant lipid species observed varied in their sphingoid long-chain base (LCB) structure with 4-hydroxydihydrosphinganine and 4t,14c-sphingediene. Other minor glycosphingolipids included sulfatides and cerebrosides with mostly sphingosine and N-linked acyl groups varying in hydroxylation and unsaturation to a 24-carbon chain (C24:1, C24 OH, C24:1 OH). These species are commonly found in white matter and are key structural components that provide stability in the multilayered myelin sheath which functions to protect and insulate the neural axons.

We found that using previous methodologies to profile multiple samples in succession resulted in varying degrees of carryover, which affected quantitation, caused retention shifts, and degraded the general analysis.^35^ For example, a 1 μL injection of a 1 mg/μL tissue sample showed carryover in ten subsequent blanks which is depicted in the Supporting Information (Figure S1A). To eliminate the carryover, we used a series of valve switches with a C-8 trap column to fractionate the sphingolipids, preventing hydrophilic and hydrophobic contaminants from being introduced to the analytical column (Figure S1B). This enrichment strategy was validated using a pool of sphingolipid standards over a range of injection volumes which showed consistent elution times and linearly correlated responses (Figure S2). Instrumental duplicate injections for the brain tissue sample produced an average percent relative standard deviation (%RSD) of 11.5% for all compounds above 0.1% relative abundance. Biological triplicates of the TIB-190 cell line generated an average %RSD of 18.4% for all compounds >0.1% relative abundance.

### Mass Spectrometric Analysis of Sphingolipids

The identification of individual sphingolipids (glycosphingolipids and sphingomyelin) employed a combination of tools for putative molecular structure assignment including (1) the known biosynthetic pathways, which reduces the number of possible structures, (2) collision-induced dissociation (CID) fragmentation spectra with accurate mass detection, (3) unique retention times, (4) and neuraminidase treatment for sialic acid linkages. A biologically informed structure list was used to initially match the intact molecular weight of compounds to detected precursor ions. This list included species with lipid structures consisting of 32–44 carbons, two to three hydroxyl groups, and up to three double bonds for all headgroups. It was observed that the charge states and associated adducts were dependent on the headgroup. For example, sphingomyelin, cerebrosides, sulfatides, and lactosylceramide primarily produced singly protonated quasimolecular ions. However, larger more complex glycosphingolipids contained multiply charged species with combinations of protons and ammonium adducts ([M + H]^+^, [M + NH_4_]^+^, [M + 2H]^2+^, [M + H + NH_4_]^2+^, [M + 2NH_4_]^2+^) which are summed to determine the compound total abundances.

**Figure 4:**
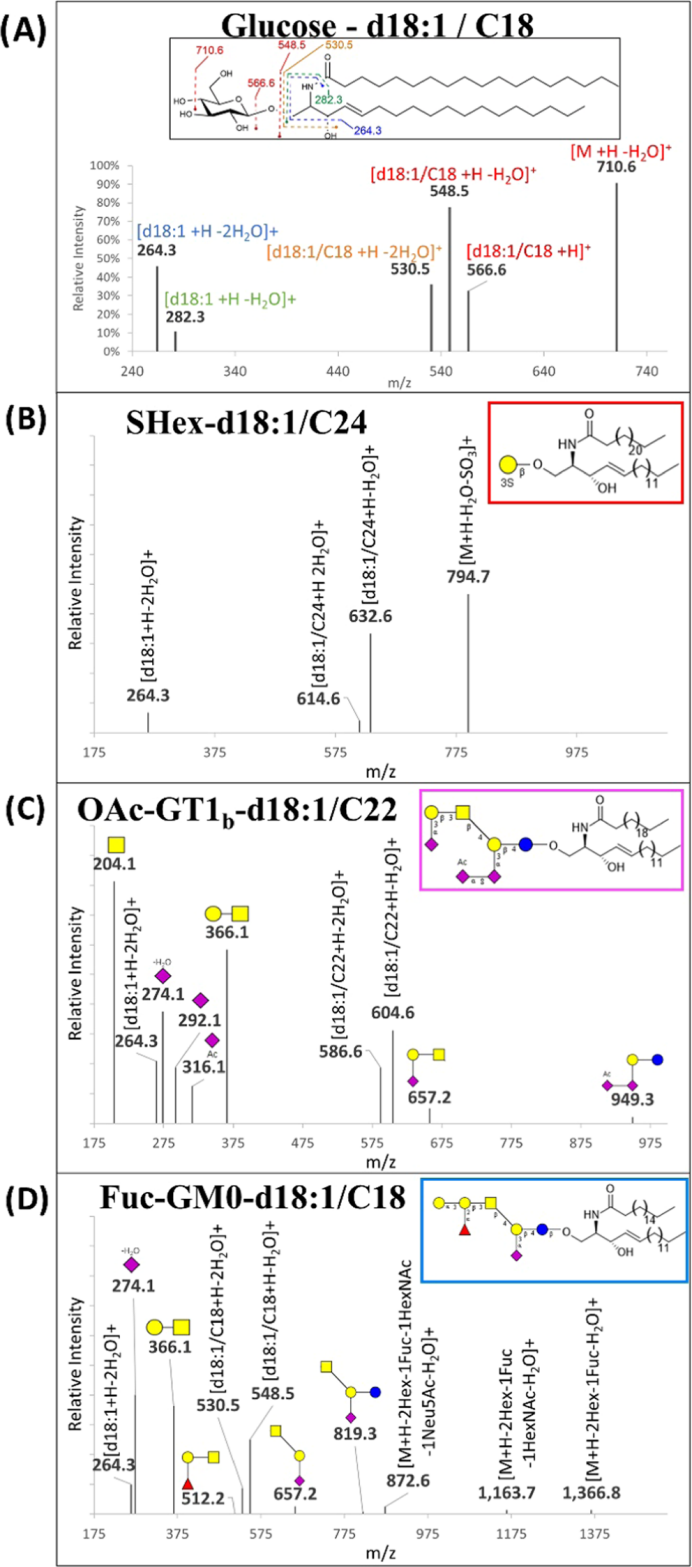
Typical fragmentations generated by CID MS/MS for compounds with various GSL headgroups including (A) Glucose-, (B) SHex-, (C) OAc-GT1_b_-, and (D) Fuc-GM0-Cer. Dominant dissociation products correspond to cleavages of glycan linkages.

The combination of various adducted species complicated the analysis by increasing the number of overlapping isobars, making CID crucial for identification. CID produced fragments corresponding to dissociation of the headgroup, the N-linked acyl, and losses of H_2_O were observed. For example, the fragmentation of a cerebroside (Hex-d18:1/C18) with a molecular weight of 727.6u was detected as a protonated species and produced fragments corresponding to H_2_O loss of the intact molecule (710.6 *m*/*z*), the ceramide (566.6, 548.5, 530.5 *m*/*z*) and the LCB (282.3, 264.3 *m*/*z*) (Figure 4A). 4t,14c-sphingediene (d18:2; 280.3, 262.3 *m*/*z*), 4-hydroxydihydrosphinganine (t18:0; 300.3, 282.3 *m*/*z*), dihydroceramide (d18:0; 302.3, 284.3 *m*/*z*), and 6-hydroxysphingosine (t18:1, 298.3, 280.3 *m*/*z*) LCBs were also discernible and imperative to distinguish hydroxyl group and double-bond positioning between the two lipid tails. Although the ceramide’s N-linked acyl and LCB moieties vary in structure, the cleavage sites shown are the most common. Additional ceramide and LCB structures and their corresponding product ions used for identification are included in the Supporting Information (Tables S1 and S2). Sulfatides (SHex-), which contain a sulfate at the C3 position of the hexose, produced a similar CID profile to cerebrosides differing in the major fragment that corresponded to the loss of both H_2_O and SO_3_ groups (Figure 4B). The presence of *N*-acetylhexosamine (HexNAc) or neuraminic acid (Neu5Ac) in large glycosphingolipids (GSLs) resulted in MS_2_ peaks with up to four linked monosaccharides. A comprehensive list of ions commonly observed from the fragmentation of the oligosaccharide headgroups was tabulated (Table 1). Neu5Ac modifications, such as acetyl or methyl groups and lactone rings, were also readily observed as product ions (Figure 4C). Glycan fragments containing fucose were also observed, for example, 1Hex1HexNAc1Fuc (512.2 *m*/*z*). However, fucose-containing compounds were not major fragments due to the labile nature of fucose under CID. Typically, fucosylated structures such as the fucosylated-GM0-d18:1/C18 were confirmed by neutral losses of terminal monosaccharides (Figure 4D).

**Table 1:** Commonly Observed Glycan Ions Produced from CID MS/MS of GSLs

glycan structure	[M + H]^+^ (*m*/*z*)	[M + H – H_2_O]^+^ (*m*/*z*)
(l)HexNAc	204.1	
(l)Neu5Ac	292.1	274.1
(l)Hex (l)HexNAc	366.1	
(l)Hex (l)Neu5Ac	454.2	
(l)Hex (l)HexNAc (l)Fuc	512.2	
(l)Hex (l)HexNAc (l)Neu5Ac	657.2	
(2)Hex (l)HexNAc (l)Neu5Ac	819.3	
(l)Hex (l)HexNAc (2)Neu5Ac	948.3	930.3
(l)Neu5Ac + OMe	307.1	
(l)Neu5Ac + OAc	334.1	316.1

Sphingomyelins (SM), a sphingolipid but not a glycolipid, containing a phosphocholine headgroup was monitored to complete the sphingolipid profile. SM species were distinct from the glycolipids in that the protonated species yielded an odd-numbered nominal mass. A common fragment corresponded to the dissociation of the phosphocholine headgroup to produce a prominent 184.1 *m*/*z* ion due to the high gas-phase basicity of the tertiary amine in the headgroup. Ceramide-1-phosphate (1P-Cer), another sphingolipid, was also present but in lower abundances.

Notably, phosphatidylcholine (PC) and phosphatidylethanolamine (PE) phospholipids are isobaric compounds observed within the elution gradient that can cause false positive identifications. Both were singly protonated, generating even nominal precursors with distinctive MS_2_ profiles. PC commonly generated a 184.1 *m*/*z* phosphocholine fragment and the neutral loss of 141 amu identified PE species. An additional source of false-positive identifications can occur from in-source fragmentation of the labile glycan headgroups. Source conditions were optimized to minimize this effect to 1% or less relative abundance. Sulfatides showed the highest degree of in-source fragmentation with loss of sulfate and produced an ion mass identical to HexCer. To a lesser extent, in-source fragmentation of sialic acid residues was observed where GD3- was initially identified as GM3-. All false positive identifications were easily distinguished and correctly identified by retention times.

### Chromatographic Behavior of Sphingolipids

**Figure 5:**
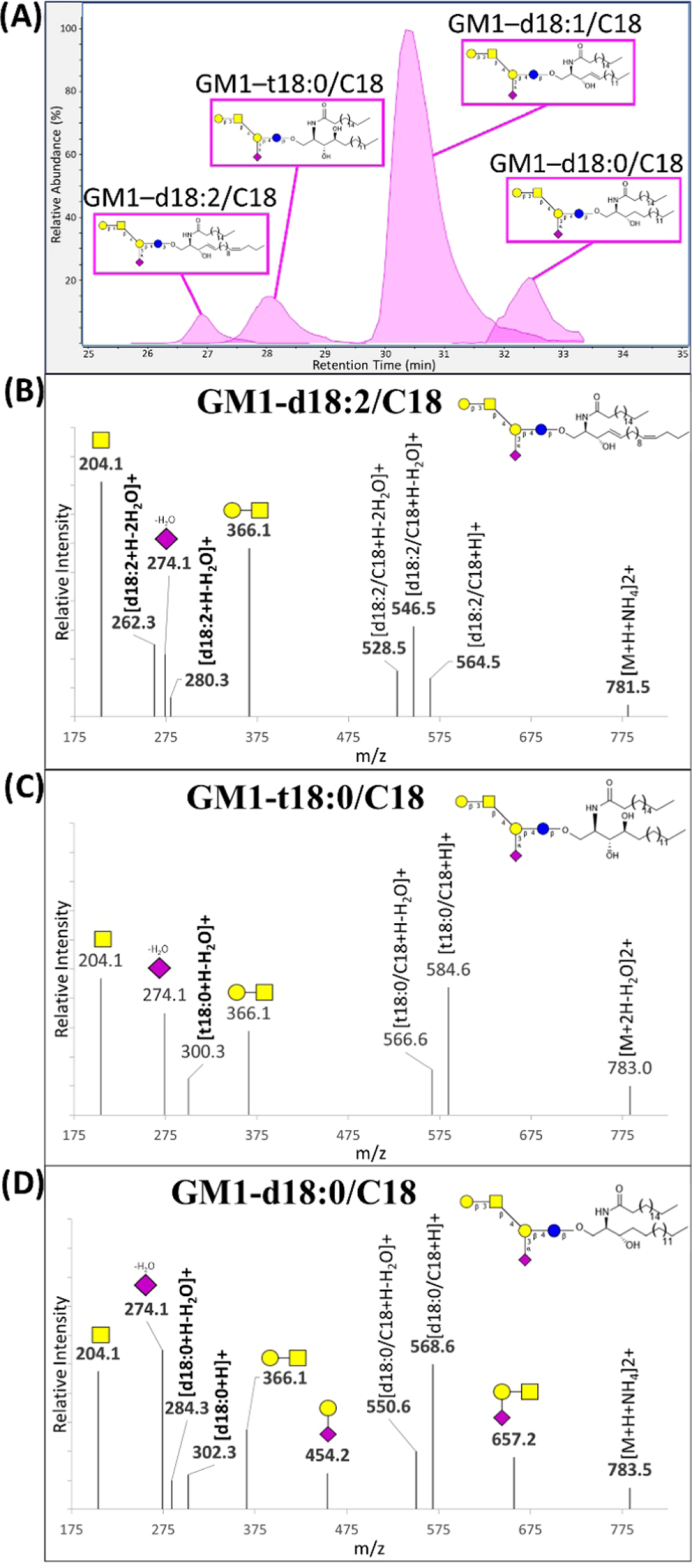
(A) Elution profile of representative LCBs. The MS/MS spectra of GM1-LCB/C18 with (B) d18:2, (C) t18:0, and (D) d18:0.

Chromatographic retention times were primarily dictated by the headgroup and the ceramides’ overall chain lengths. Sphingomyelins showed a broad lipid profile that encompassed the entire chromatogram and were used to assign relative retention time (RRT) values for species containing the same lipid structure but differing in their headgroup. Although RRT values varied depending on the specific lipid, average RRTs for all observed matching ceramides were assigned to give general headgroup-dependent elution trends. With SM (RRT 1.000) being the latest eluting, neutral GSLs showed slightly earlier elution times, and differences became more prominent with larger glycans: Hex- (0.987 RRT), Lac- (0.961), Gb3 (0.940), GA1 (0.928), Fuc-GA1 (0.925), and GA0 (0.904). Sulfated and monosialylated GSLs with their single anionic moieties eluted earlier: SHex- (0.868 RRT), SLac- (0.822), GM3 (0.861), GM2 (0.833), GM1 (0.839). The most pronounced shifts in retention were observed from polysialylated GSLs: GD3- (0.771 RRT), GD2- (0.764), GD1- (0.755), Fuc-GD1- (0.753), GT1- (0.724), and GQ1- (0.700). Differences in LCB structures were also observed chromatographically for structures with a fixed headgroup and N-linked acyl. Increasing hydroxide groups and unsaturated bonds resulted in earlier elution times (Figure 5A), which was confirmed by CID fragmentation (Figure 5B–D). Isomeric lipids differing in double bond position were resolved where ceramides containing sphingosine and an unsaturated fatty acid (d18:1/FA:1) eluted earlier than 4t,14c-sphingediene with a saturated acyl group (d18:2/FA) (Figure S3).

The identity of some structures that yielded only partially informative CID profiles were further confirmed by the linear correlation of chromatographic retention times to acyl chain length (Figure S4). The more abundant species of a group (same headgroup and LCB) that contained complete CID spectra were used to identify the less abundant sphingolipids by matching the observed retention times to the expected values.

### Neuraminidase Treatments for Structural Analysis of Gangliosides

Reverse-phase chromatography could not separate glycan isomers with multiple neuraminic acid linkages and positions. For this reason, we employed α2–3,6,8-neuraminidases to determine the sialic acid linkages in GT1_b_, GD1_a_, and GM1_a_. Surprisingly, upon α2–3,6,8 neuraminidase treatment, the completely desialylated ganglioside core GA1 was not observed. Experimental data showed cleavage of sialic acid residues attached to the terminal galactose, but α2–3 and α2–8 sialic acids linked to the first core galactose remained intact. Molecular dynamic simulations were performed and corroborated the experimental data showing a steric hindrance of the neuraminidase active site by the terminal galactose when the tetrasaccharide core was present (Figure S5). By accounting for this feature, glycan structures were determined with a single enzyme treatment.

### Automated and Extrapolated Compound ID

We sought to automate the structural identification of all sphingolipids (including all glycolipids) using both the accurate mass, MS_2_ (CID), and chromatographic elution patterns. We therefore developed an in-house spectral library containing identified structures including the molecular formula, characteristic charge state, adducts, CID product ions, and retention times using Agilent’s Personal Compound Database and Library (PCDL, B08.00) software. This workflow is especially useful when conducting studies with large sample sets as manual verification of MS_2_ spectra is tedious and requires a notable degree of user experience. For reference, a generated library score of ≥10 indicated a correct identification, lower scores required further investigation. Although the use of this software greatly expedites the identification of sphingolipids, it is currently limited to compounds that have been manually verified previously. For this reason, initial identification of compounds using the comprehensive CSV database of monoisotopic masses was necessary.

Following the identification and validation of all sphingolipid species observed, the compound list for each sample was exported as an individual CSV file. An in-house Python script was then used that read the exported files, created a running dictionary for each unique structure, and reorganized the abundances from all samples into a single spreadsheet. The processed data was then analyzed in Microsoft excel and heatmaps were generated for visualization of the sphingolipid profiles. This method was applied on biological samples described below.

### GSL Profile of Biological Samples in Serum and Cells

**Figure 6:**
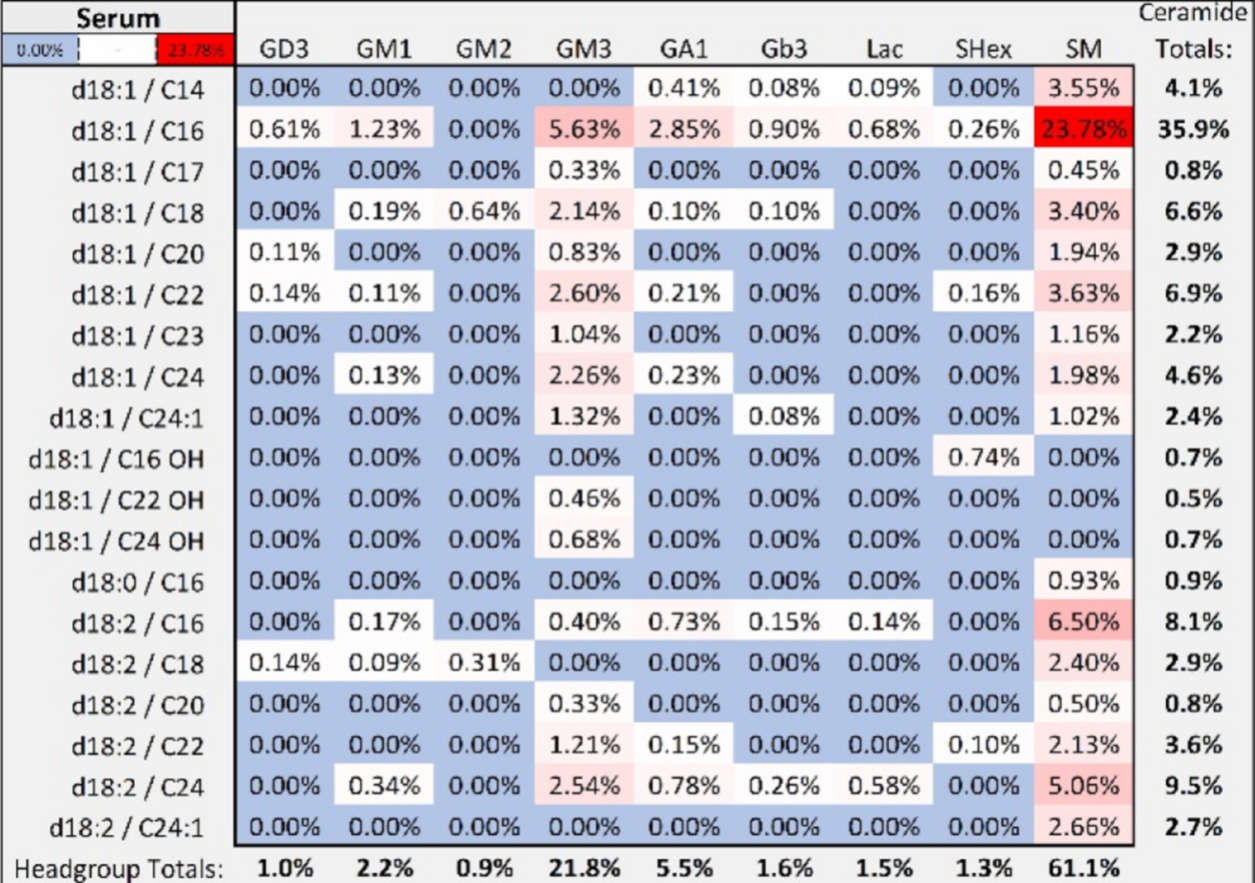
Heatmap summarizing observed sphingolipids (≥0.01% relative abundances) in human serum.

A sphingolipid profile was generated from a commercial human serum pool and used as a quality control to monitor the sample preparation and instrument suitability (Figure S6). Of the 78 compounds observed in the serum profile, the most abundant glycosphingolipid was GM3-d18:1/16, a truncated core ganglioside (Figure 6). The major source of structural diversity observed in serum can be attributed to the lipid moiety as most of the glycan headgroups contained only one to three monosaccharide residues. GSLs in serum are thought to come from the shedding of membranes from tissues that circulate in micelles and lipoprotein complexes. We have previously found GSLs to be also bound to HDL and other lipoprotein particles.^36^ We further examined GSLs in cell lines. We profiled CESS (TIB-190) cells, an immortalized line commonly used to study T-cells. The profile showed comparable results to that of serum in both the major headgroup and lipid structures, suggesting T-cells and serum shared many common GSLs (Figure S7). The compound list for all samples with relative abundances and nomenclature details were tabulated (Table S4).

## Conclusions

A robust reverse-phase nanoflow HPLC-Q-ToF method was developed for profiling glycosphingolipids (and other sphingolipids) from human brain tissue, serum, and a lymphoblastic cell line. This method was developed to address the typical issues common to sphingolipid analysis such as carry-over and false-positive identifications. By utilizing previously discovered human biosynthetic pathways and correlating structures to fragmentation patterns along with excellent chromatographic reproducibility, exact structures can be assigned with a high degree of confidence. Future work that would greatly benefit the field of sphingolipids would include the development of software that can utilize the identification tools described in this work.

## Supporting Information Available

Additional experimental details; materials; and methods as mentioned in the text including the instrumental configuration for the reported method (PDF)
Python3 script used for organization of processed sample files (ZIP)
