# Supplementary material for: Profiling Intact Glycosphingolipids with Automated Structural Annotation and Quantitation from Human Samples with Nanoflow Liquid Chromatography Mass Spectrometry
Source: Anal Chem. 2024 Apr 2;96(15):5951–9. doi: 10.1021/acs.analchem.4c00077 (PMC11024888; doi:10.1021/acs.analchem.4c00077)
Supplement: Supplementary file 1 — ac4c00077_si_001.pdf [file ac4c00077_si_001.pdf]

Supporting Information

Profiling Intact Glycosphingolipids with automated structural annotation and quantitation from human samples with Nanoflow Liquid Chromatography Mass Spectrometry

Ryan L. Schindler[1], Armin Oloumi[1], Jennyfer Tena[1], Michael Russelle S. Alvarez[1], Yiyun Liu[1], Sheryl Grijaldo[1], Mariana Barboza[2], Lee-way Jin[3], Angela M. Zivkovic[4], Carlito B. Lebrilla[1]*

[1]Department of Chemistry, University of California, Davis, California, 95616, USA.

[2]Innovation Institute for Food and Health, University of California, Davis, California, 95616, USA.

[3]Department of Pathology and Laboratory Medicine, University of California Davis Medical Center, Sacramento, California, 95817, USA.

[4]Department of Nutrition, University of California, Davis, California, 95616, USA.

Corresponding Author:

*E-mail: cblebrilla@ucdavis.edu.

Table of Contents

Figure S1 – S7: Figures as mentioned in the text.
Figure S8 – S9: Instrument configuration figures.
Table S1 – S2: Glycosphingolipid fragments observed from collision-induced dissociation as mentioned in the text.
Table S3: HPLC capillary configuration table.
Table S4: Full compound list for human brain tissue, human serum, and TIB-190 cells.

Figure S1. Total compound chromatograms carryover comparison after the injection of a pooled tissue sample at a concentration of 1mg initial tissue weight / µL of solvent A) Neural tissue sample injection and the subsequent ten blank injections using the ChipCube system with methodology from reference 35. B) Neural tissue sample injection with no detectable compounds in the subsequent blank injection using the reported instrumental configuration and method.

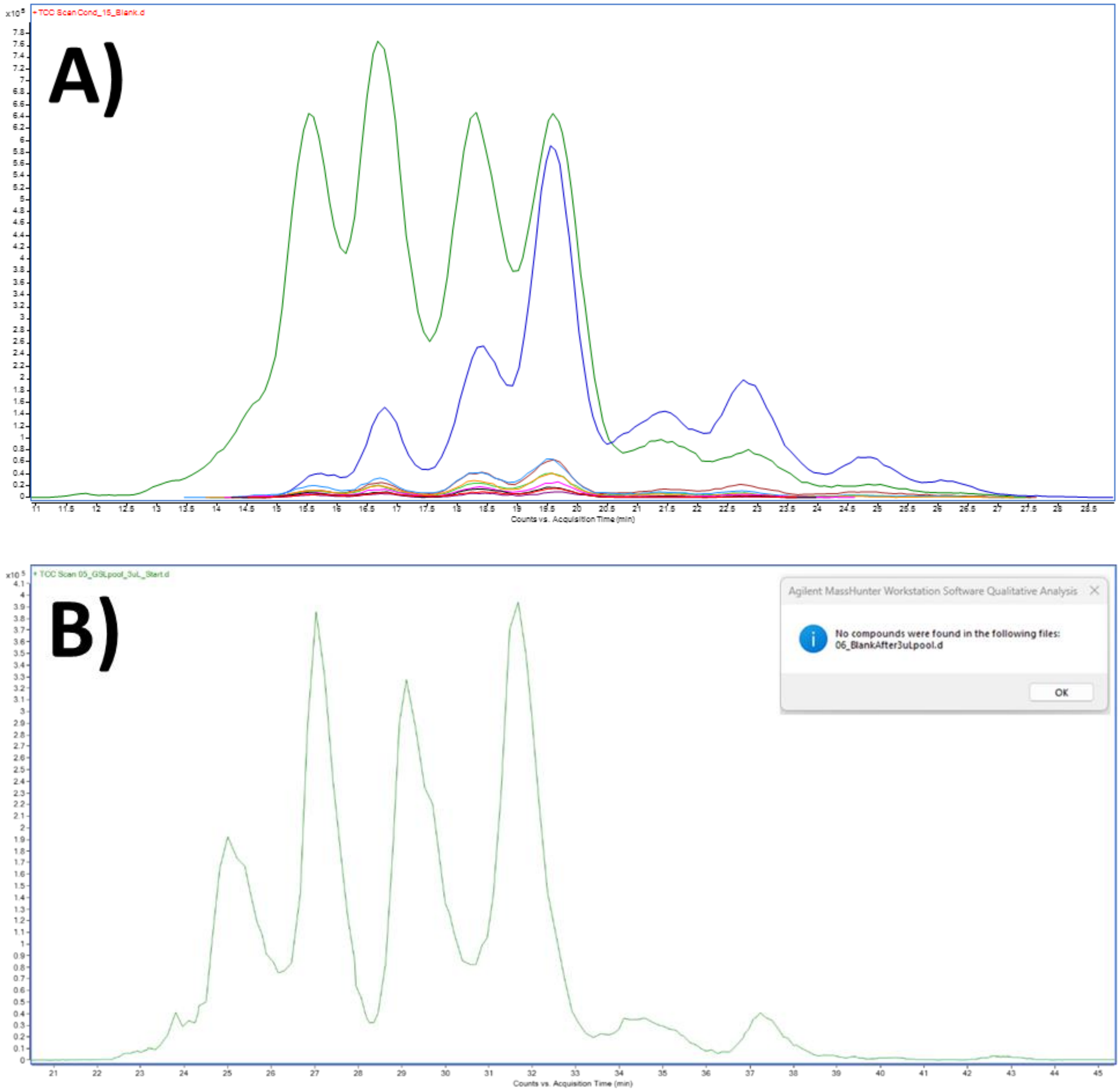

Figure S2. Overlaid chromatograms of a standard pool with 0.5µL, 1.0µL, and 1.5µL injections and the corresponding calibration curve. Note that GM3 and SM4 had similar concentrations and responses, resulting in an overlap in the calibration curve.

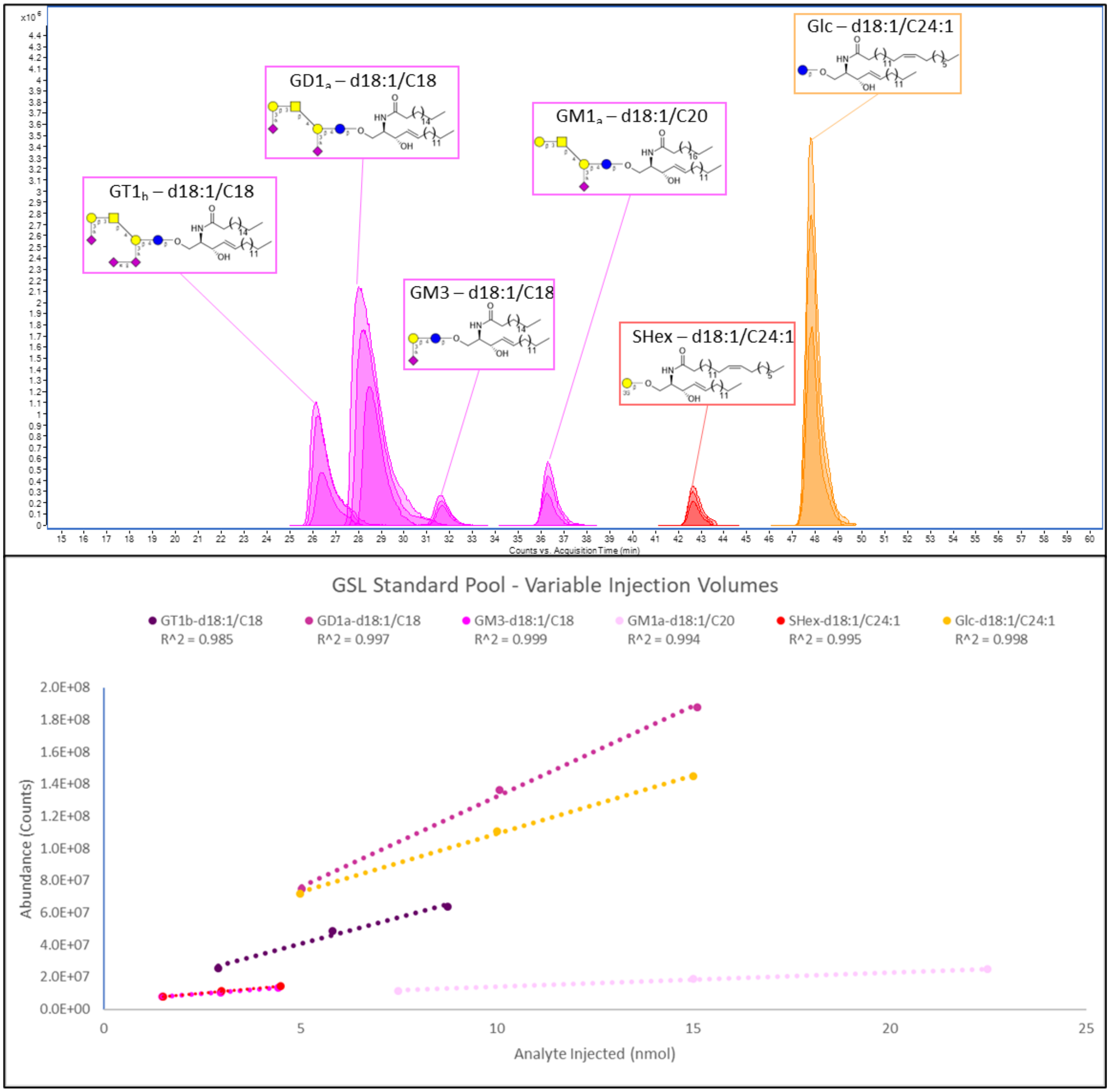

Figure S3. Separation of isomeric sphingolipids differing in double bond position.

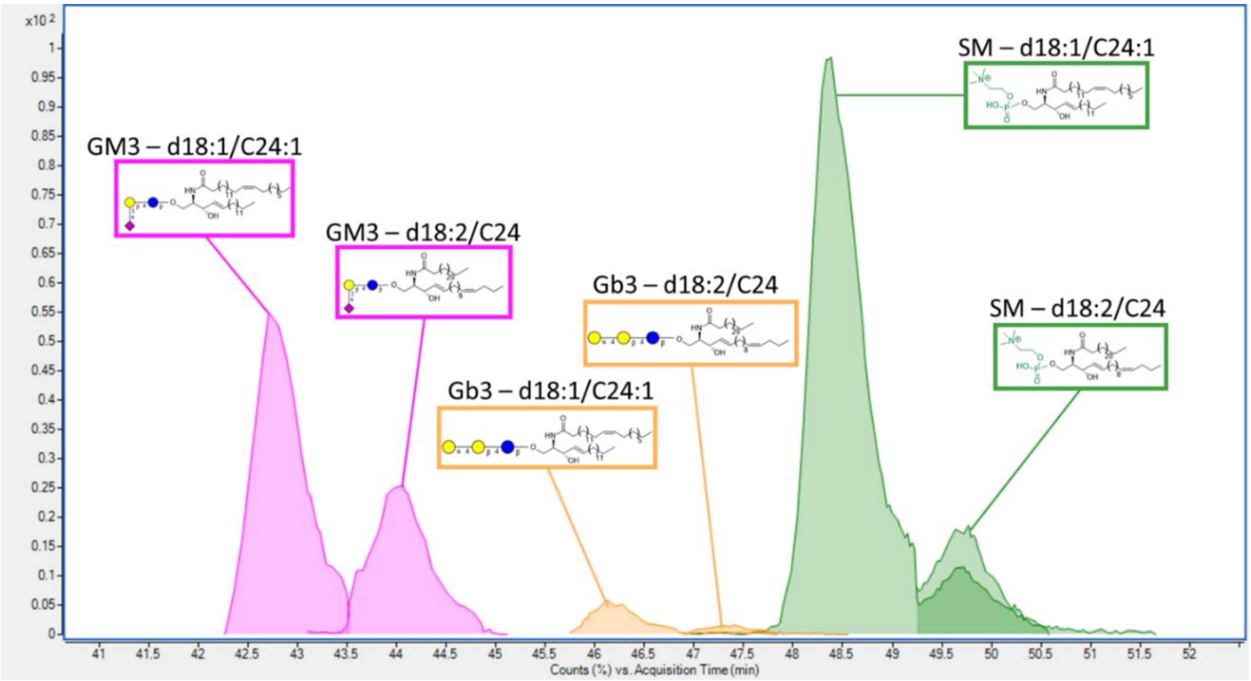

Figure S4. Sphingomyelin- and GD1–d18:1/FA species observed in human brain tissue and the correlation between total lipid carbon number and retention time.

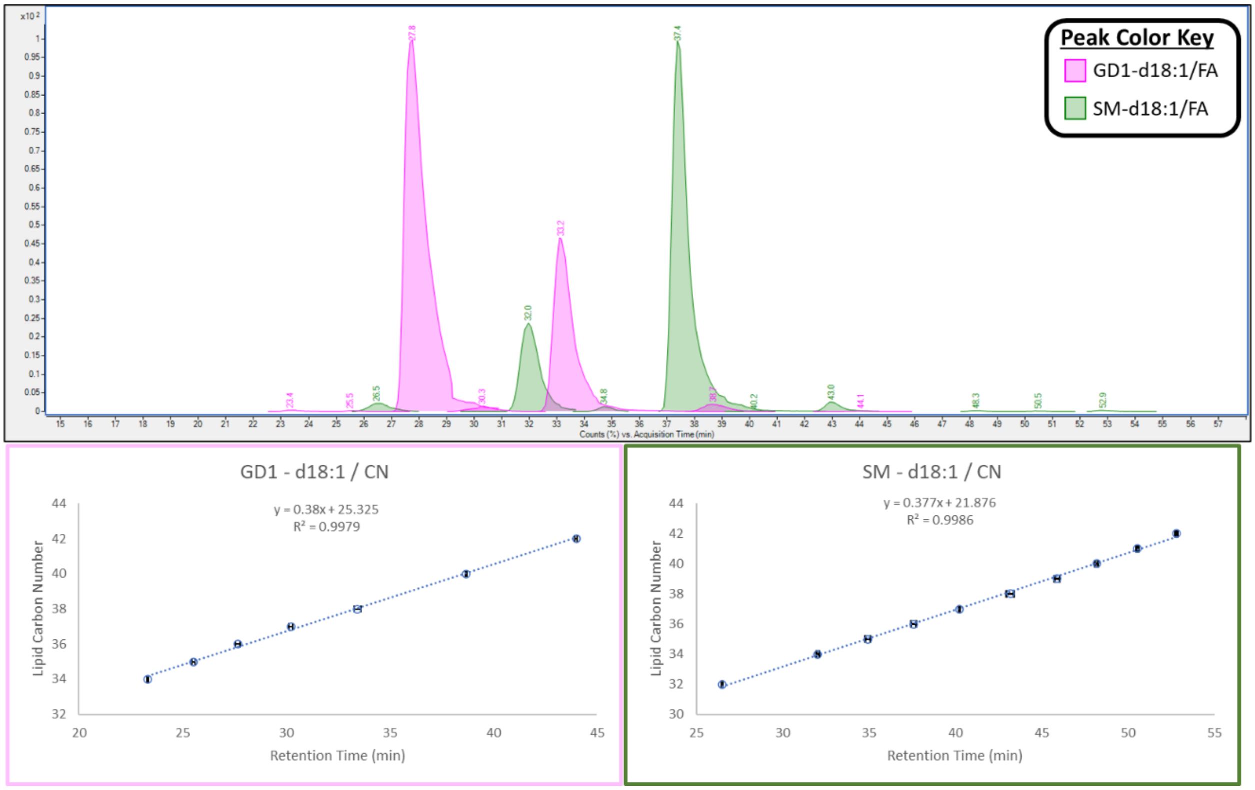

Figure S5. Computational depiction of α2-3,6,8 neuraminidase, from Clostridium perfringens, with active site interactions with GM1a. Sialic acid in GM1a is accessible to active site residue Asp62 but is being blocked by terminal galactose from Arg37 and Arg245.

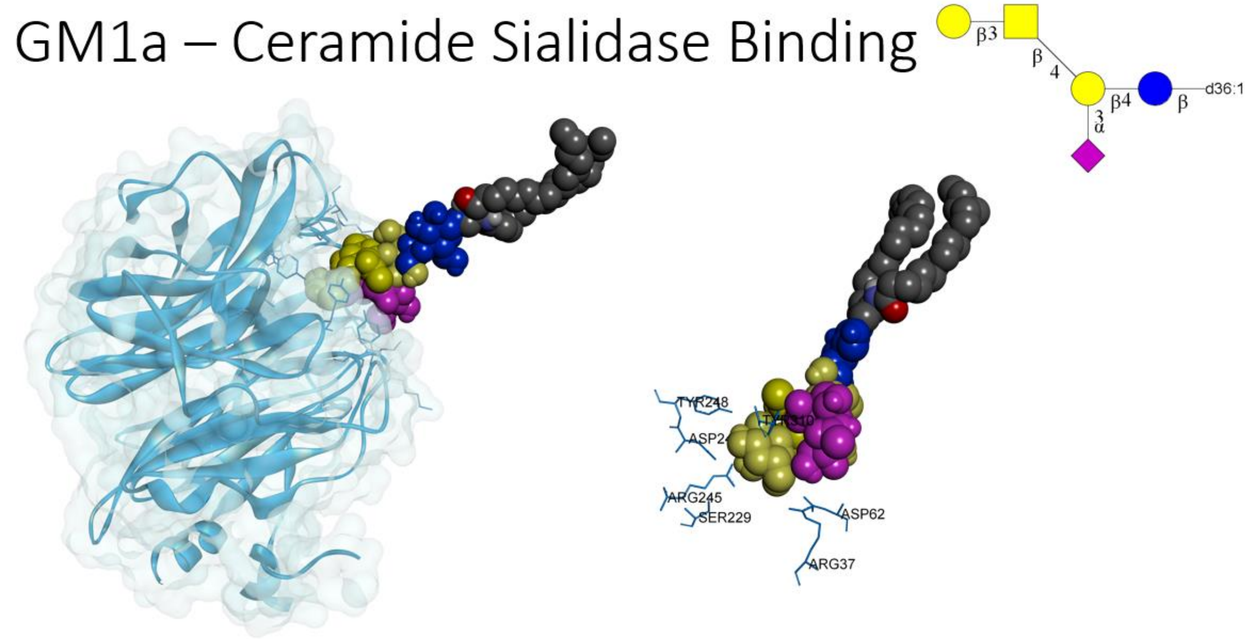

Figure S6. Example chromatogram of human serum sphingolipid profile with 25 of 78 compounds depicted.

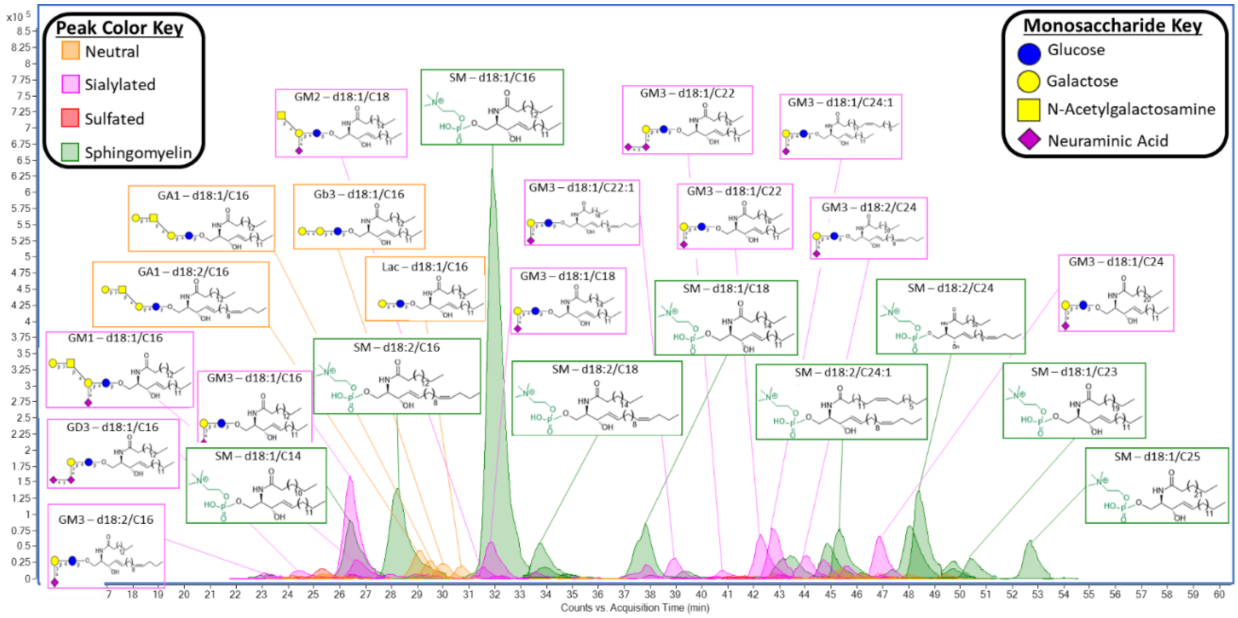

Figure S7. Summary heatmap profile of T-cells (TIB-190) for all sphingolipids ≥0.01% relative abundance.

**TIB-190 Cells** (0.00% - 22.56%)	GD1	GD3	GM2	GM3	GA1	Gb3	SLac	Lac	SM	Ceramide Totals:
d18:1 / C14	0.00%	0.44%	0.00%	1.63%	0.00%	0.10%	0.00%	0.61%	3.07%	**5.9%**
d18:1 / C16	1.36%	5.56%	0.70%	12.31%	1.09%	1.95%	0.94%	3.87%	22.56%	**50.3%**
d18:1 / C18	0.00%	0.43%	0.00%	1.09%	0.00%	0.11%	0.00%	0.33%	0.00%	**2.0%**
d18:1 / C20	0.00%	0.40%	0.00%	2.59%	0.00%	0.00%	0.00%	0.23%	0.00%	**3.2%**
d18:1 / C22	0.00%	0.44%	0.00%	3.22%	0.00%	0.00%	0.06%	0.50%	0.00%	**4.2%**
d18:1 / C24	0.00%	0.69%	0.00%	4.66%	0.00%	0.00%	0.00%	0.35%	0.00%	**5.7%**
d18:1 / C22:1	0.00%	0.00%	0.00%	1.13%	0.00%	0.00%	0.00%	0.00%	0.00%	**1.1%**
d18:1 / C24:1	0.41%	2.01%	0.00%	13.10%	0.00%	0.16%	0.19%	0.73%	1.11%	**17.7%**
d18:1 / C26:1	0.00%	0.00%	0.00%	1.64%	0.00%	0.00%	0.00%	0.00%	0.00%	**1.6%**
d18:0 / C16	0.00%	0.00%	0.00%	4.57%	0.00%	0.00%	0.00%	1.32%	0.00%	**5.9%**
d18:2 / C16	0.00%	0.00%	0.00%	0.22%	0.00%	0.00%	0.00%	0.00%	0.79%	**1.0%**
t18:0 / C24:1	0.00%	0.00%	0.00%	0.37%	0.00%	0.00%	0.00%	0.00%	0.00%	**0.4%**
Headgroup Totals:	**1.8%**	**10.0%**	**0.7%**	**46.5%**	**1.1%**	**2.3%**	**1.2%**	**7.9%**	**27.5%**	

Figure S8. Active HPLC modules and 10pt/2ps µ-switching valve configuration.

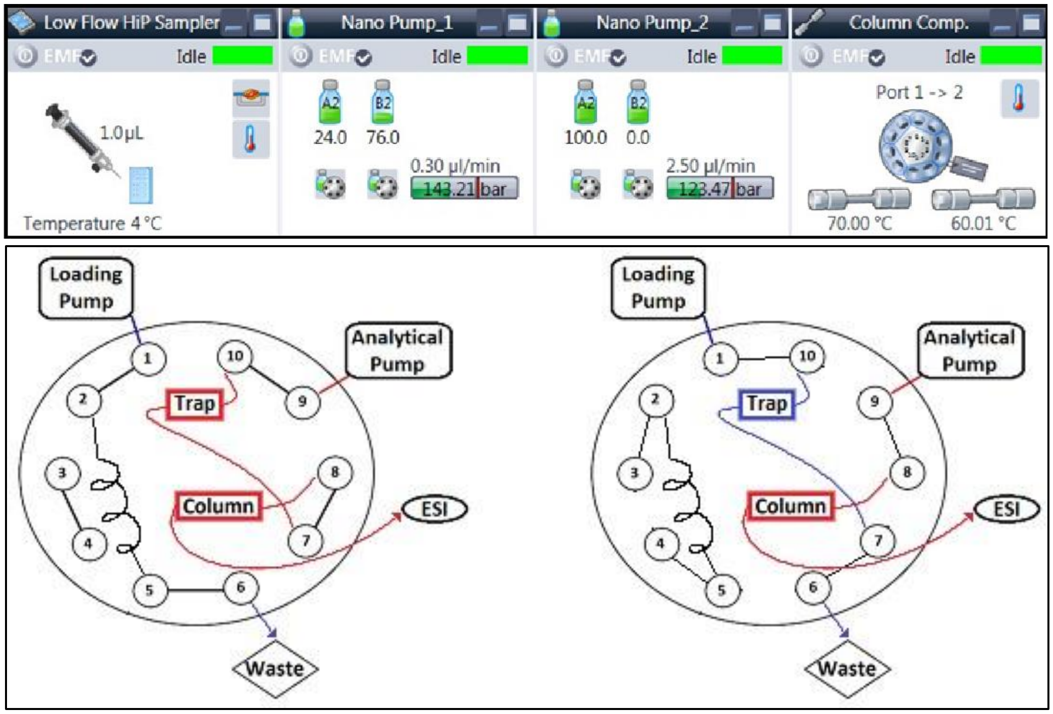

Figure S9. Nanoflow ESI source (Agilent Technologies, G1992A) nebulizer positioning, spray trajectory, and corresponding reference mass response.

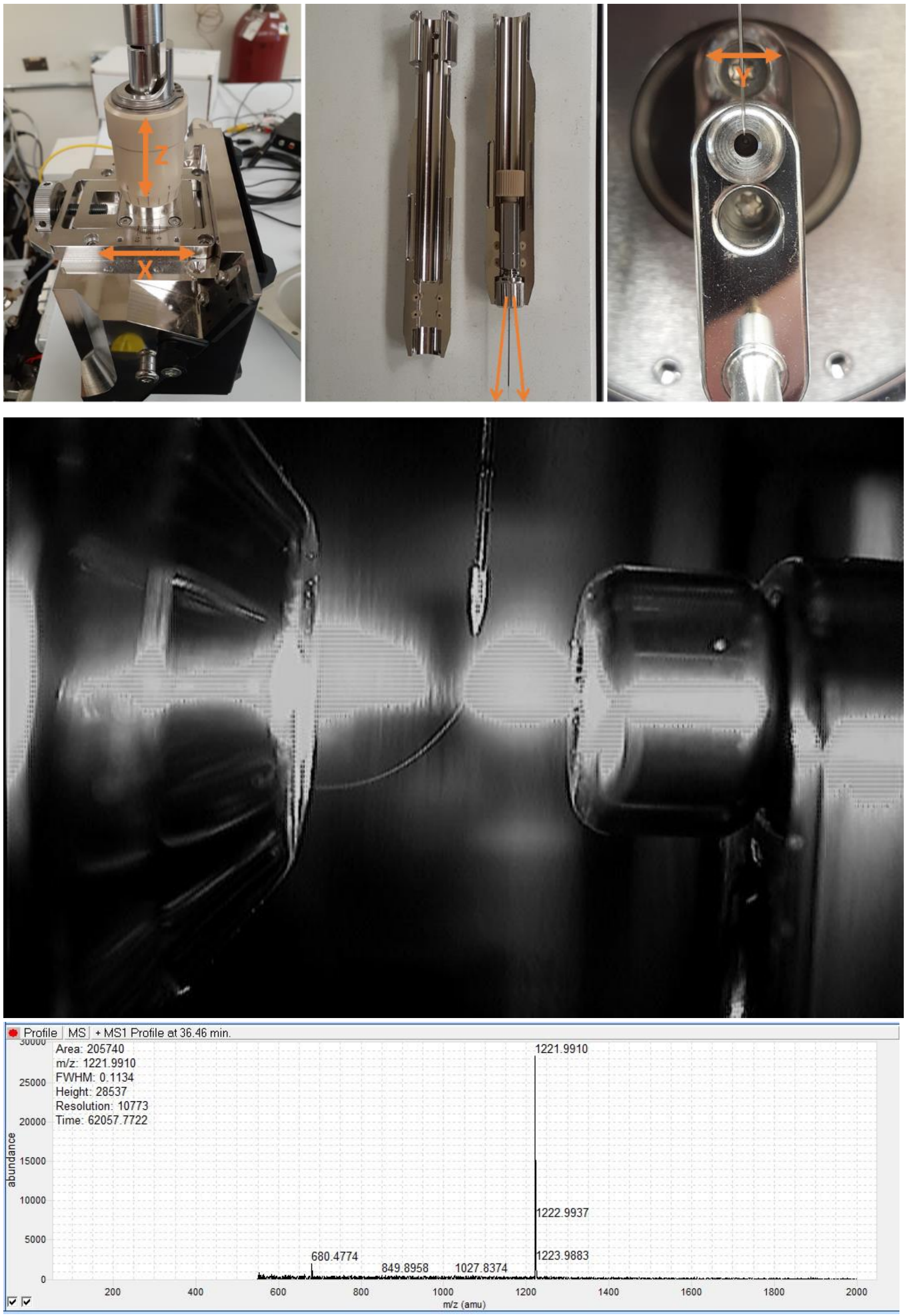

Table S1. Common ceramide fragment ions from headgroup dissociation.

Hydroxyl Groups[1]	Double bonds	Carbon Number	[Cer+H]+	[Cer+H-H2O]+	[Cer+H-2xH2O]+
d	1	32	510.488619	492.478054	474.467489
		33	524.504269	506.493704	488.483139
		34	538.519919	520.509354	502.498789
		35	552.535569	534.525004	516.514439
		36	566.551219	548.540654	530.530089
		37	580.566869	562.556304	544.545739
		38	594.582519	576.571954	558.561389
		39	608.598169	590.587604	572.577039
		40	622.613819	604.603254	586.592689
		41	636.629469	618.618904	600.608339
		42	650.645119	632.634554	614.623989
		43	664.660769	646.650204	628.639639
		44	678.676419	660.665854	642.655289
t	0	32	528.499184	510.488619	492.478054
		34	556.530484	538.519919	520.509354
		36	584.561784	566.551219	548.540654
		38	612.593084	594.582519	576.571954
		40	640.624384	622.613819	604.603254
		41	654.640034	636.629469	618.618904
		42	668.655684	650.645119	632.634554
		43	682.671334	664.660769	646.650204
		44	696.686984	678.676419	660.665854

[1]The number of hydroxyl groups are assigned with letters, d=2, t=3

Table S2. Common Long-chain base fragments from headgroup and N-linked acyl dissociation.

Hydroxyl Groups[1]	Double bonds	Carbon Number	[LCB+H]+	[LCB+H-H2O]+	[LCB+H-2xH2O]+
d	0	18	302.305904	284.295339	266.284774
	1	18	300.290254	282.279689	264.269124
	2	18	298.274604	280.264039	262.253474
	0	20	330.337204	312.326639	294.316074
	1	20	328.321554	310.310989	292.300424
	2	20	326.305904	308.295339	290.284774
t	0	18	318.300819	300.290254	282.279689
	1	18	316.285169	298.274604	280.264039

[1]The number of hydroxyl groups are assigned with letters, d=2, t=3

Table S3. HPLC capillary configuration.

Nanoflow 1200 Series HPLC - Connecting Capillaries						
**Module**	**From (fitting)**	**To (fitting)**	**pn**	**ID (mm)**	**Length (mm)**	**Dwell Volume (µL)**
**Nano Pump - Loading (0.1 - 4 µL/min) pn: G2226A**	EMPV (WPF)	Flow Sensor (WPF)	G1375-87321	0.025	220	0.108
	Flow Sensor (MP)	µWPS Valve, port 1 (MP)	G1375-87322	0.025	350	0.172
**Nano Pump - Analytical (0.1 - 4 µL/min) pn: G2226A**	EMPV (WPF)	Flow Sensor (WPF)	G1375-87321	0.025	220	0.108
	Flow Sensor (MP)	µTCC Valve, port 9 (MP)	G1375-87322	0.025	350	0.172
**Micro WPS pn: G1377A + Thermostat pn: 1330B**	µAS Valve		0101-1050	Internal Channel		0.07
	µWPS, port 1 (MP)	Metering Device (WPF)	-			
	Sample Loop (8µL)		G1375-87315	-		
	µWPS-Needle		G1377-87201	-		
	µWPS-Needle Seat		G1377-87001	-		
	µWPS, port 6 (MP)	µTCC, port 1 (MP)	5067-1582	µ-Inline Filter		0.46
**TCC pn: G1316C**	10pt/2ps µ-Switching Valve		5067-4144	Internal Channel		0.089
	Port 10 (MP)	Trap Column (WPF)	G1375-87322	0.025	350	0.172
	Trap Column (WPF)	Port 7 (MP)	G1375-87320	0.025	100	0.049
	Port 2 (MP)	Port 5 (MP)	G1375-87320	0.025	100	0.049
	Port 8 (MP)	Analytical Column Inlet (WPF)	G1375-87320	0.025	100	0.049
**Nano ESI pn: G1992A**	Analytical Column Outlet (WPF)	nESI (Nebulizer)	G4240-87300	0.015	900	0.159
**Fittings**	**Acronym**	**Part Number**	**Description**			
	MP	5065-4410	PEEK fitting (Metric M4)			
	WPF	5065-4422	Double winged PEEK nut & ferrule			

Table S4. Full compound list with relative abundances of each sample type.

Intact Sphingolipid[1]	Headgroup	Sphingoid Base	Acyl Group	Monoisotopic Mass	Tissue (%)	Serum (%)	TIB-190 (%)
*3_1_0_4_0_0_d18:1 / C20	Acidic GQ1	d18:1	20	2447.1928	0.24%	0.00%	0.00%
3_1_0_4_0_0_d18:1 / C18	Acidic GQ1	d18:1	18	2419.1598	0.09%	0.00%	0.00%
3_1_0_3_0_0_d18:1 / C22	Acidic GT1	d18:1	22	2184.1295	0.13%	0.00%	0.00%
3_1_0_3_0_0_t18:0 / C22	Acidic GT1	t18:0	22	2202.1406	0.07%	0.00%	0.00%
3_1_0_3_0_0_d18:1 / C21	Acidic GT1	d18:1	21	2170.1109	0.03%	0.00%	0.00%
3_1_0_3_0_0_d18:2 / C20	Acidic GT1	d18:2	20	2154.0878	0.03%	0.00%	0.00%
*3_1_0_3_0_0_d18:1 / C20	Acidic GT1	d18:1	20	2156.0974	3.96%	0.00%	0.00%
3_1_0_3_0_0_t18:0 / C20	Acidic GT1	t18:0	20	2174.1096	0.76%	0.00%	0.00%
3_1_0_3_0_0_d18:0 / C20	Acidic GT1	d18:0	20	2158.1095	0.28%	0.00%	0.00%
3_1_0_3_0_0_d18:1 / C19	Acidic GT1	d18:1	19	2142.0865	0.06%	0.00%	0.00%
*3_1_0_3_0_0_d18:1 / C18	Acidic GT1	d18:1	18	2128.0672	1.94%	0.05%	0.00%
3_1_0_3_0_0_t18:0 / C18	Acidic GT1	t18:0	18	2146.0766	0.21%	0.00%	0.00%
3_1_0_3_0_0_d18:0 / C18	Acidic GT1	d18:0	18	2130.0793	0.14%	0.00%	0.00%
3_1_0_3_0_0_t18:1 / C16	Acidic GT1	t18:1	16	2116.0046	0.04%	0.00%	0.00%
2_0_0_3_0_0_d18:2 / C20	Acidic GT3	d18:2	20	1788.9133	0.01%	0.00%	0.00%
3_1_0_2_0_0_d18:1 / C26:1	Acidic GD1	d18:1	26:1	1947.09	0.03%	0.00%	0.00%
3_1_0_2_0_0_d18:1 / C24:1	Acidic GD1	d18:1	24:1	1919.053	0.07%	0.00%	0.93%
3_1_0_2_0_0_d18:1 / C24	Acidic GD1	d18:1	24	1921.0727	0.03%	0.00%	0.00%
3_1_0_2_0_0_d18:1 / C22	Acidic GD1	d18:1	22	1893.0363	0.45%	0.00%	0.00%
3_1_0_2_0_0_t18:0 / C22	Acidic GD1	t18:0	22	1911.0468	0.07%	0.00%	0.00%
3_1_0_2_0_0_d18:2 / C20	Acidic GD1	d18:2	20	1862.9912	0.34%	0.00%	0.00%
*3_1_0_2_0_0_d18:1 / C20	Acidic GD1	d18:1	20	1865.0071	10.31%	0.00%	0.00%
3_1_0_2_0_0_t18:0 / C20	Acidic GD1	t18:0	20	1883.0156	1.76%	0.24%	0.00%
3_1_0_2_0_0_d18:1 / C19	Acidic GD1	d18:1	19	1850.9945	0.31%	0.00%	0.00%
3_1_0_2_0_0_d18:2 / C18	Acidic GD1	d18:2	18	1834.961	0.20%	0.00%	0.00%
*3_1_0_2_0_0_d18:1 / C18	Acidic GD1	d18:1	18	1836.9769	23.63%	0.29%	0.00%
3_1_0_2_0_0_t18:0 / C18	Acidic GD1	t18:0	18	1854.9862	1.99%	0.00%	0.00%
3_1_0_2_0_0_d18:1 / C17	Acidic GD1	d18:1	17	1822.9522	0.04%	0.00%	0.00%
3_1_0_2_0_0_d18:1 / C16	Acidic GD1	d18:1	16	1808.9453	0.07%	0.00%	2.55%
3_1_0_2_0_0_t18:0 / C16	Acidic GD1	t18:0	16	1826.9292	0.03%	0.00%	0.00%
*2_1_0_2_0_0_d18:1 / C20	Acidic GD2	d18:1	20	1702.9626	0.26%	0.00%	0.00%
2_1_0_2_0_0_d18:0 / C20	Acidic GD2	d18:0	20	1720.9809	0.06%	0.00%	0.00%
2_1_0_2_0_0_d18:1 / C18	Acidic GD2	d18:1	18	1674.9236	0.98%	0.00%	0.00%
2_1_0_2_0_0_t18:0 / C18	Acidic GD2	t18:0	18	1692.9472	0.08%	0.00%	0.00%
2_1_0_2_0_0_d18:0 / C18	Acidic GD2	d18:0	18	1676.945	0.08%	0.00%	0.00%
2_0_0_2_0_0_d18:1 / C24:1	Acidic GD3	d18:1	24:1	1553.9211	0.00%	0.00%	2.95%
2_0_0_2_0_0_d18:1 / C24	Acidic GD3	d18:1	24	1555.9395	0.00%	0.00%	1.05%
2_0_0_2_0_0_d18:1 / C22	Acidic GD3	d18:1	22	1527.913	0.00%	0.15%	0.92%
*2_0_0_2_0_0_d18:1 / C20	Acidic GD3	d18:1	20	1499.8681	0.36%	0.11%	0.68%

2_0_0_2_0_0_d18:1 / C19	Acidic GD3	d18:1	19	1485.8592	0.02%	0.00%	0.00%
2_0_0_2_0_0_d18:2 / C18	Acidic GD3	d18:2	18	1469.8387	0.05%	0.14%	0.00%
2_0_0_2_0_0_d18:1 / C18	Acidic GD3	d18:1	18	1471.8361	0.24%	0.00%	0.68%
2_0_0_2_0_0_t18:0 / C18	Acidic GD3	t18:0	18	1489.8558	0.24%	0.00%	0.00%
2_0_0_2_0_0_d18:1 / C16	Acidic GD3	d18:1	16	1443.8143	0.03%	0.61%	0.00%
2_0_0_2_0_0_d18:1 / C14	Acidic GD3	d18:1	14	1415.7873	0.00%	0.00%	0.76%
4_1_0_1_0_0_d18:0 / C18	Acidic GM0	d18:0	18	1709.9591	0.02%	0.00%	0.00%
3_1_0_1_0_0_d18:2 / C24	Acidic GM1	d18:2	24	1627.9598	0.00%	0.34%	0.00%
3_1_0_1_0_0_d18:1 / C24	Acidic GM1	d18:1	24	1629.9697	0.00%	0.13%	0.00%
3_1_0_1_0_0_d18:1 / C22	Acidic GM1	d18:1	22	1601.9482	0.09%	0.11%	0.00%
3_1_0_1_0_0_d18:2 / C20	Acidic GM1	d18:2	20	1571.9044	0.14%	0.00%	0.00%
3_1_0_1_0_0_t18:0 / C20	Acidic GM1	t18:0	20	1591.9225	0.25%	0.00%	0.00%
3_1_0_1_0_0_d18:2 / C18	Acidic GM1	d18:2	18	1543.8654	0.53%	0.09%	0.00%
*3_1_0_1_0_0_d18:1 / C18	Acidic GM1	d18:1	18	1545.8816	11.57%	0.19%	0.00%
3_1_0_1_0_0_t18:0 / C18	Acidic GM1	t18:0	18	1563.8908	1.41%	0.00%	0.00%
3_1_0_1_0_0_d18:0 / C18	Acidic GM1	d18:0	18	1547.8948	0.51%	0.00%	0.00%
3_1_0_1_0_0_d18:1 / C17	Acidic GM1	d18:1	17	1531.8666	0.04%	0.00%	0.00%
3_1_0_1_0_0_d18:2 / C16	Acidic GM1	d18:2	16	1515.8409	0.00%	0.17%	0.00%
3_1_0_1_0_0_d18:1 / C16	Acidic GM1	d18:1	16	1517.8511	0.13%	1.23%	0.00%
2_1_0_1_0_0_d18:1 / C20	Acidic GM2	d18:1	20	1411.8568	0.07%	0.00%	0.00%
2_1_0_1_0_0_t18:0 / C20	Acidic GM2	t18:0	20	1429.8767	0.03%	0.00%	0.00%
2_1_0_1_0_0_d18:2 / C18	Acidic GM2	d18:2	18	1381.8151	0.07%	0.31%	0.00%
2_1_0_1_0_0_d18:1 / C18	Acidic GM2	d18:1	18	1383.8264	0.98%	0.64%	0.00%
2_1_0_1_0_0_t18:0 / C18	Acidic GM2	t18:0	18	1401.8396	0.17%	0.00%	0.00%
2_1_0_1_0_0_d18:1 / C16	Acidic GM2	d18:1	16	1355.8091	0.00%	0.00%	0.87%
2_0_0_1_0_0_d18:1 / C24:1	Acidic GM3	d18:1	26:1	1290.8586	0.00%	0.00%	1.88%
2_0_0_1_0_0_d18:1 / C24:1	Acidic GM3	d18:1	24:1	1262.8403	0.05%	3.84%	16.36%
2_0_0_1_0_0_t18:0 / C24:1	Acidic GM3	t18:0	24:1	1280.8387	0.00%	0.68%	0.71%
2_0_0_1_0_0_d18:1 / C24	Acidic GM3	d18:1	24	1264.8518	0.00%	2.27%	6.34%
2_0_0_1_0_0_d18:2 / C23	Acidic GM3	d18:2	23	1248.8156	0.00%	0.09%	0.00%
2_0_0_1_0_0_t18:0 / C23:1	Acidic GM3	t18:0	23:1	1266.8166	0.00%	0.17%	0.00%
2_0_0_1_0_0_d18:1 / C23	Acidic GM3	d18:1	23	1250.8287	0.00%	1.04%	0.00%
2_0_0_1_0_0_d18:2 / C22	Acidic GM3	d18:2	22	1234.8062	0.00%	1.21%	0.00%
2_0_0_1_0_0_t18:0 / C22:1	Acidic GM3	t18:0	22:1	1252.8078	0.00%	0.46%	0.00%
2_0_0_1_0_0_d18:1 / C22:1	Acidic GM3	d18:1	22:1	1234.7953	0.00%	0.00%	1.12%
2_0_0_1_0_0_d18:1 / C22	Acidic GM3	d18:1	22	1236.8129	0.00%	2.60%	7.20%
*2_0_0_1_0_0_d18:2 / C20	Acidic GM3	d18:2	20	1206.7714	0.00%	0.33%	0.00%
2_0_0_1_0_0_d18:1 / C20	Acidic GM3	d18:1	20	1208.7952	0.00%	0.83%	5.90%
2_0_0_1_0_0_d18:1 / C18	Acidic GM3	d18:1	18	1180.7471	1.23%	2.14%	1.70%
2_0_0_1_0_0_d18:1 / C17	Acidic GM3	d18:1	17	1166.7296	0.00%	0.33%	0.00%
2_0_0_1_0_0_d18:2 / C16	Acidic GM3	d18:2	16	1150.7055	0.00%	0.40%	0.00%
2_0_0_1_0_0_d18:1 / C16	Acidic GM3	d18:1	16	1152.7209	0.04%	5.63%	12.82%
2_0_0_1_0_0_d18:0 / C16	Acidic GM3	d18:0	16	1154.7261	0.00%	0.00%	1.15%

2_0_0_1_0_0_d18:1 / C14	Acidic GM3	d18:1	14	1124.6821	0.00%	0.00%	1.24%
*3_1_1_2_0_0_d18:1 / C20	Acidic Fuc-GD1	d18:1	20	2011.0637	0.07%	0.00%	0.00%
3_1_1_2_0_0_d18:1 / C18	Acidic Fuc-GD1	d18:1	18	1983.0376	0.14%	0.00%	0.00%
4_1_1_1_0_0_d18:1 / C22	Acidic Fuc-GM0	d18:1	22	1910.0368	0.02%	0.00%	0.00%
4_1_1_1_0_0_d18:1 / C20	Acidic Fuc-GM0	d18:1	20	1882.0313	0.93%	0.00%	0.00%
4_1_1_1_0_0_d18:1 / C18	Acidic Fuc-GM0	d18:1	18	1854.0015	2.50%	0.11%	0.00%
3_1_1_1_0_0_d18:1 / C18	Acidic Fuc-GM1	d18:1	18	1691.9393	0.17%	0.00%	0.00%
3_1_1_1_0_0_t18:0 / C18	Acidic Fuc-GM1	t18:0	18	1709.9591	0.02%	0.00%	0.00%
4_1_1_0_0_0_d18:2 / C20	Neutral Fuc-GA0	d18:2	20	1588.9393	0.04%	0.00%	0.00%
*4_1_1_0_0_0_d18:1 / C20	Neutral Fuc-GA0	d18:1	20	1590.9386	0.30%	0.00%	0.00%
4_1_1_0_0_0_t18:0 / C20	Neutral Fuc-GA0	t18:0	20	1608.9579	0.10%	0.00%	0.00%
4_1_1_0_0_0_d18:1 / C16	Neutral Fuc-GA0	d18:1	16	1534.8774	0.00%	0.17%	0.00%
3_1_1_0_0_0_d18:1 / C18	Neutral Fuc-GA1	d18:1	18	1400.8421	0.02%	0.00%	0.00%
3_1_1_0_0_0_d18:1 / C16	Neutral Fuc-GA1	d18:1	16	1372.8151	0.00%	0.14%	0.00%
3_1_0_0_0_0_d18:1 / C24:1	Neutral GA1	d18:1	24:1	1336.8588	0.00%	0.78%	0.00%
3_1_0_0_0_0_d18:1 / C24	Neutral GA1	d18:1	24	1338.8754	0.00%	0.23%	0.00%
3_1_0_0_0_0_d18:2 / C22	Neutral GA1	d18:2	22	1308.8268	0.00%	0.15%	0.00%
3_1_0_0_0_0_d18:1 / C22	Neutral GA1	d18:1	22	1310.8415	0.00%	0.21%	0.00%
3_1_0_0_0_0_d18:0 / C20	Neutral GA1	d18:0	20	1284.8047	0.00%	0.12%	0.00%
3_1_0_0_0_0_d18:1 / C18	Neutral GA1	d18:1	18	1254.7805	0.04%	0.10%	0.00%
3_1_0_0_0_0_d18:2 / C16	Neutral GA1	d18:2	16	1224.7366	0.00%	0.73%	0.00%
3_1_0_0_0_0_d18:1 / C16	Neutral GA1	d18:1	16	1226.7551	0.00%	2.85%	0.78%
3_1_0_0_0_0_d18:1 / C14	Neutral GA1	d18:1	14	1198.7188	0.00%	0.42%	0.00%
3_0_0_0_0_0_d18:2 / C24	Neutral Gb3	d18:2	24	1133.7817	0.00%	0.34%	0.00%
3_0_0_0_0_0_d18:1 / C18	Neutral Gb3	d18:1	18	1051.7056	0.00%	0.10%	0.00%
3_0_0_0_0_0_d18:2 / C16	Neutral Gb3	d18:2	16	1021.6558	0.00%	0.15%	0.00%
3_0_0_0_0_0_d18:1 / C16	Neutral Gb3	d18:1	16	1023.6721	0.00%	0.90%	1.88%
3_0_0_0_0_0_d18:1 / C14	Neutral Gb3	d18:1	14	995.6473	0.00%	0.08%	0.00%
2_0_0_0_0_1_d18:1 / C14	Sulfate SLac	d18:1	16	941.5769	0.00%	0.00%	1.56%
2_0_0_0_0_0_d18:1 / C24:1	Neutral Lac	d18:1	24:1	971.7291	0.02%	0.58%	0.92%
2_0_0_0_0_0_d18:1 / C24	Neutral Lac	d18:1	24	973.7404	0.00%	0.00%	0.42%
2_0_0_0_0_0_d18:1 / C22	Neutral Lac	d18:1	22	945.7196	0.00%	0.00%	0.51%
2_0_0_0_0_0_d18:1 / C20	Neutral Lac	*d18:1	20	917.6686	0.00%	0.00%	0.44%
2_0_0_0_0_0_d18:1 / C18	Neutral Lac	d18:1	18	889.6497	0.07%	0.00%	0.59%
2_0_0_0_0_0_d18:2 / C16	Neutral Lac	d18:2	16	859.6033	0.00%	0.14%	0.00%
2_0_0_0_0_0_d18:1 / C16	Neutral Lac	d18:1	16	861.6189	0.00%	0.68%	5.82%
2_0_0_0_0_0_d18:1 / C14	Neutral Lac	d18:1	14	833.5967	0.00%	0.09%	0.58%
1_0_0_0_0_0_d18:2 / C26 OH	Neutral Hex	d18:2	26 OH	853.7017	0.06%	0.00%	0.00%
1_0_0_0_0_0_d18:2 / C25 OH	Neutral Hex	d18:2	25 OH	839.679	0.07%	0.00%	0.00%
1_0_0_0_0_0_d18:2 / C24 OH	Neutral Hex	d18:2	24 OH	825.6701	0.28%	0.00%	0.00%
1_0_0_0_0_0_d18:1 / C24:1	Neutral Hex	d18:1	24:1	809.6768	0.13%	0.37%	0.00%
1_0_0_0_0_0_t18:0 / C24:1	Neutral Hex	t18:0	24:1	827.6878	0.17%	0.00%	0.00%
1_0_0_0_0_0_t18:0 / C22:1	Neutral Hex	t18:0	22:1	799.6521	0.07%	0.00%	0.00%

1_0_0_0_0_0_d18:1 / C18	Neutral Hex	d18:1	18	727.5984	0.05%	0.00%	0.00%
1_0_0_0_0_1_d18:1 / C26:1 OH	Sulfate Shex	d18:1	26:1 OH	933.6579	0.25%	0.00%	0.00%
1_0_0_0_0_1_d18:1 / C26:1	Sulfate Shex	d18:1	26:1	917.663	0.15%	0.00%	0.00%
1_0_0_0_0_1_d18:1 / C26 OH	Sulfate Shex	d18:1	26 OH	935.674	0.04%	0.00%	0.00%
1_0_0_0_0_1_d18:1 / C25:1 OH	Sulfate Shex	d18:1	25:1 OH	919.6428	0.23%	0.00%	0.00%
1_0_0_0_0_1_d18:1 / C25:1	Sulfate Shex	d18:1	25:1	903.6463	0.26%	0.00%	0.00%
1_0_0_0_0_1_d18:1 / C25 OH	Sulfate Shex	d18:1	25 OH	921.6599	0.14%	0.00%	0.00%
1_0_0_0_0_1_d18:1 / C25	Sulfate Shex	d18:1	25	905.6598	0.05%	0.00%	0.00%
1_0_0_0_0_1_d18:1 / C24:1 OH	Sulfate Shex	d18:1	24:1 OH	905.6275	0.59%	0.27%	0.00%
1_0_0_0_0_1_d18:1 / C24:1	Sulfate Shex	d18:1	24:1	889.6323	0.59%	0.00%	0.00%
1_0_0_0_0_1_d18:1 / C24 OH	Sulfate Shex	d18:1	24 OH	907.6434	0.47%	0.00%	0.00%
1_0_0_0_0_1_d18:1 / C24	Sulfate Shex	d18:1	24	891.6464	0.09%	0.00%	0.00%
1_0_0_0_0_1_d18:1 / C23:1	Sulfate Shex	d18:1	23:1	875.6035	0.01%	0.00%	0.00%
1_0_0_0_0_1_t18:0 / C24	Sulfate Shex	t18:0	24	909.6566	0.01%	0.00%	0.00%
1_0_0_0_0_1_d18:1 / C23 OH	Sulfate Shex	d18:1	23 OH	893.6275	0.20%	0.00%	0.00%
1_0_0_0_0_1_d18:1 / C23	Sulfate Shex	d18:1	23	877.6321	0.03%	0.00%	0.00%
1_0_0_0_0_1_d18:1 / C22:1	Sulfate Shex	d18:1	22:1	861.5771	0.00%	0.10%	0.00%
1_0_0_0_0_1_d18:1 / C22 OH	Sulfate Shex	d18:1	22 OH	879.6076	0.09%	0.16%	0.00%
1_0_0_0_0_1_d18:2 / C18	Sulfate Shex	d18:2	18	805.541	0.05%	0.00%	0.00%
1_0_0_0_0_1_d18:1 / C18 OH	Sulfate Shex	d18:1	18 OH	823.5509	0.05%	0.00%	0.00%
1_0_0_0_0_1_d18:1 / C18	Sulfate Shex	d18:1	18	807.5502	0.16%	0.00%	0.00%
1_0_0_0_0_1_d18:1 / C16 OH	Sulfate Shex	d18:1	16 OH	795.5245	0.00%	0.74%	0.00%
1_0_0_0_0_1_d18:1 / C16	Sulfate Shex	d18:1	16	779.5219	0.05%	0.26%	0.00%
SM_d18:2 / C26	Sphingomyelin	d18:2	26	840.7087	0.04%	0.00%	0.00%
SM _d18:2 / C25	Sphingomyelin	d18:2	25	826.693	0.05%	0.00%	0.00%
SM _d18:2 / C24:1	Sphingomyelin	d18:2	24:1	810.6593	0.00%	2.67%	0.00%
SM _d18:1 / C25	Sphingomyelin	d18:1	25	828.7147	0.00%	0.06%	0.00%
SM _d18:1 / C24:1	Sphingomyelin	d18:1	24:1	812.6776	0.64%	6.09%	0.59%
SM _t18:0 / C24	Sphingomyelin	t18:0	24	830.6878	0.05%	0.00%	0.00%
SM _d18:1 / C24	Sphingomyelin	d18:1	24	814.6891	0.06%	1.98%	0.00%
SM _d18:2 / C23	Sphingomyelin	d18:2	23	798.6524	0.02%	0.31%	0.00%
SM _d18:1 / C23	Sphingomyelin	d18:1	23	800.6699	0.03%	1.17%	0.00%
SM _d18:2 / C22	Sphingomyelin	d18:2	22	784.643	0.00%	2.13%	0.00%
SM _d18:1 / C22	Sphingomyelin	d18:1	22	786.662	0.04%	3.64%	0.00%
SM _d18:2 / C21	Sphingomyelin	d18:2	21	770.6207	0.00%	0.25%	0.00%
SM _t18:0 / C22	Sphingomyelin	t18:0	22	804.6576	0.04%	0.00%	0.00%
SM _d18:2 / C20	Sphingomyelin	d18:2	20	756.6018	0.00%	0.50%	0.00%
SM _d18:1 / C20	Sphingomyelin	d18:1	20	758.6305	0.43%	1.94%	0.00%
SM _d18:1 / C19	Sphingomyelin	d18:1	19	744.6154	0.04%	0.00%	0.00%
SM _d18:2 / C18	Sphingomyelin	d18:2	18	728.5848	1.57%	2.40%	0.00%
SM _d18:1 / C18	Sphingomyelin	d18:1	18	730.6006	15.89%	3.40%	0.00%
SM _d18:0 / C18	Sphingomyelin	d18:0	18	732.6116	0.18%	0.07%	0.00%
SM _d18:1 / C17	Sphingomyelin	d18:1	17	716.5847	0.19%	0.45%	0.00%

SM _d18:2 / C16	Sphingomyelin	d18:2	16	700.5522	0.12%	6.51%	0.00%
SM _d18:1 / C16	Sphingomyelin	d18:1	16	702.5696	4.22%	23.81%	15.98%
SM _t18:0 / C16	Sphingomyelin	t18:0	16	720.5814	0.03%	0.00%	0.00%
SM _d18:1 / C16	Sphingomyelin	d18:1	16	704.5828	0.06%	0.93%	0.00%
SM _d18:2 / C14	Sphingomyelin	d18:2	14	672.5275	0.00%	0.37%	0.00%
SM _d18:1 / C14	Sphingomyelin	d18:1	14	674.5384	0.46%	3.56%	2.11%
1. Database nomenclature: Hex_HexNAc_Fuc_Neu5Ac_Neu5Gc_Sulf_(OH#)CN:unsat/CN:unsat (OH#)							
*A mixture of Sphingoid bases were observed containing both 18 (major) and 20 (minor) carbon length LCBs.							
