# Supplementary material for: Profiling Intact Glycosphingolipids with Automated Structural Annotation and Quantitation from Human Samples with Nanoflow Liquid Chromatography Mass Spectrometry
Source: Anal Chem. 2024 Apr 2;96(15):5951–9. doi: 10.1021/acs.analchem.4c00077 (PMC11024888; doi:10.1021/acs.analchem.4c00077)
Supplement: Supplementary file 2 — ac4c00077_si_002.zip [file ac4c00077_si_002.zip › Python3 Script/INSTRUCTIONS for Python3 Script.docx]

Instructions for using this Python script to organize sphingolipid data from MassHunter Qualitative Analysis:

After all compounds for each datafile have been verified, ensure that the compound table includes the following columns in the following order:

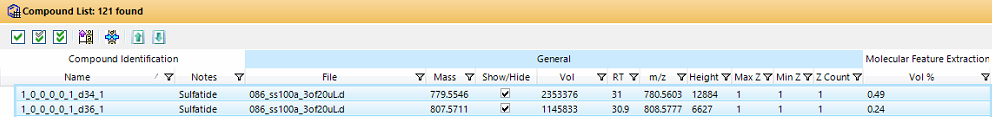


Export each file individually by highlighting all compounds and right-clicking a compound > Export…

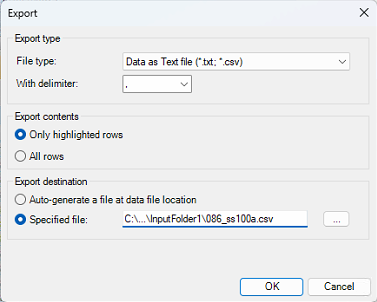


Ensure Python 3 is installed and run the script to generate individual CSV files in “OutputFolder1” and the consolidated CSV file (PythonOutput_Worklist_AbsAbund.csv) that includes data from all individual files.
